# Vesicular Stomatitis Virus Transmission Dynamics Within Its Endemic Range in Chiapas, Mexico

**DOI:** 10.3390/v16111742

**Published:** 2024-11-06

**Authors:** Lawrence H. Zhou, Federico Valdez, Irene Lopez Gonzalez, Willian Freysser Urbina, Ariadna Ocaña, Cristell Tapia, Armando Zambrano, Edilberto Hernandez Solis, Debra P. C. Peters, Chad E. Mire, Roberto Navarro, Luis L. Rodriguez, Kathryn A. Hanley

**Affiliations:** 1 Department of Biology, New Mexico State University, Las Cruces, NM 88003, USA; khanley@nmsu.edu; 2 United States Department of Agriculture, Agricultural Research Services, Plum Island Animal Disease Center and National Bio- and Agro-Defense Facility, Manhattan, KS 66502, USA; federico.valdez@usda.gov (F.V.); luisvsv@gmail.com (L.L.R.); 3 United States Department of Agriculture, Agricultural Research Services, National Bio and Agro-Defense Facility, Foreign Arthropod-Borne Animal Diseases Research Unit, Manhattan, KS 66502, USA; chad.mire@usda.gov; 4 Servicio Nacional de Sanidad, Inocuidad y Calidad Agroalimentaria (SENASICA), Ciudad de México 29020, Chiapas, Mexico; irene.gonzalez@senasica.gob.mx; 5 Instituto Interamericano de Cooperación para la Agricultura (IICA)—Laboratorio de Biología Molecular LBS2 Tuxtla Gutiérrez, Ciudad de México 29020, Chiapas, Mexico; mvzurbina_86@hotmail.com (W.F.U.); ariadna.ocana.i@senasica.gob.mx (A.O.); cristell.tapia.i@senasica.gob.mx (C.T.); armando.zambrano.i@senasica.gob.mx (A.Z.); hersoed.16@hotmail.com (E.H.S.); 6 Office of National Programs and the SCINet Big Data Program, United States Department of Agriculture, Beltsville, MD 20705, USA; deb.peters@usda.gov; 7 Comisión México-Estados Unidos para la Prevención de la Fiebre Aftosa y otras Enfermedades Exóticas de los Animales (CPA), Mexico City 64590, Mexico State, Mexico

**Keywords:** vesicular stomatitis virus, Ceratopogonidae, Culicidae, Psychodidae, Simuliidae, endemic transmission, arbovirus

## Abstract

Vesicular stomatitis virus (VSV), comprising vesicular stomatitis New Jersey virus (VSNJV) and vesicular stomatitis Indiana virus (VSIV), emerges from its focus of endemic transmission in Southern Mexico to cause sporadic livestock epizootics in the Western United States. A dearth of information on the role of potential arthropod vectors in the endemic region hampers efforts to identify factors that enable endemicity and predict outbreaks. In a two-year, longitudinal study at five cattle ranches in Chiapas, Mexico, insect taxa implicated as VSV vectors (blackflies, sandflies, biting midges, and mosquitoes) were collected and screened for VSV RNA, livestock vesicular stomatitis (VS) cases were monitored, and serum samples were screened for neutralizing antibodies. VS cases were reported during the rainy (*n* = 20) and post-rainy (*n* = 2) seasons. Seroprevalence against VSNJV in adult cattle was very high (75–100% per ranch) compared with VSIV (0.6%, all ranches). All four potential vector taxa were sampled, and VSNJV RNA was detected in each of them (11% VSNJV-positive of 874 total pools), while VSIV RNA was only detected in four pools of mosquitoes. Our findings indicate that VSNJV is the dominant serotype across our sampling sites with a variety of potential insect vectors involved in its transmission throughout the year. Although no livestock cases were reported in Chiapas during the dry season, VSNJV was detected in insects during this period, suggesting that mechanisms other than transmission from livestock support VSV endemicity.

## 1. Introduction

Vesicular stomatitis New Jersey virus (VSNJV) and vesicular stomatitis Indiana virus (VSIV) (genus *Vesiculovirus*, family *Rhabdoviridae*), herein generalized as VSV, are the agents of vesicular stomatitis (VS) in livestock [1,2,3,4]. VSV is thought to be primarily transmitted by arthropod vectors, although direct transmission occurs within livestock herds during epizootics [2,5]. The geographic ranges of VSNJV and VSIV overlap and together span North and South America [1,2,6,7,8]. Both viruses are endemic to Southern Mexico and Central America, where livestock cases are reported annually, and multiple lineages of each virus co-circulate [9,10,11,12]. Additionally, VSNJV and VSIV both make sporadic incursions northward from the endemic range at two-to-eight-year intervals, resulting in epizootic outbreaks in northern Mexico and the United States (USA) that can last up to three years [1,2,6,13,14,15]. Similar epizootic outbreaks have also been documented in northern South America [2].

VS affects all hoofed livestock species, with negative effects on animal weight and production [16,17,18,19]. Cases are primarily reported in cattle, horses, and swine, and although VS is self-limiting and rarely fatal, symptoms manifest as painful lesions with high viral titers in the mouth, tongue, udders, teats, coronary bands, and feet [1,2,16,17,20]. VS outbreaks in the USA trigger quarantines and disrupt livestock movement and trade [6,16,21]. In ruminants and swine, VS symptoms are clinically indistinguishable from foot-and-mouth disease (FMD), a fatal viral disease eradicated from the USA in 1929 [1,7,17]. This clinical similarity necessitates the reporting of VS cases to animal health authorities across the Americas and complicates FMD surveillance efforts in countries working to eradicate FMD or maintain FMD-free status [13,16,17,22].

Why VSV sustains continuous transmission in Southern Mexico and Central America but cannot accomplish this farther north or south of this region remains a mystery [1,14]. Hypotheses include requirements for specific environments, vectors, or wildlife reservoir hosts, all three of which may be required in synchrony [23,24,25]. To date, the limited number of studies of VSV transmission ecology make it difficult to test these hypotheses. Across its epizootic range, species in the families Simuliidae (black flies); Psychodidae, genus *Lutzomyia* spp. (sand flies); Ceratopogonidae, genus *Culicoides* spp. (biting midges); and Culicidae (mosquitoes) have been implicated as vectors of VSV via field-collected insects and experimental vector competence studies [26,27,28,29,30,31,32,33,34]. Black flies have been shown experimentally to effectively transmit VSV to several livestock species [27,35,36,37,38,39], and VSV has been detected in field-collected black flies and *Culicoides* spp. biting midges during VSV epizootics in the Western and Midwestern USA (e.g., Utah, Colorado, Kansas, and New Mexico) [28,29,40,41,42,43,44,45,46]. From the 1980s to 1990s, *Lutzomyia* spp. sandflies were incriminated as the main VSV vector in sustained VSV transmission on Ossabaw Island, Georgia, USA, but they have not been linked to a VSV epizootic outbreak in the USA since that time [47,48,49]. VSV was isolated from mosquitoes in an epizootic in New Mexico, USA, in 1965, and experimental studies have demonstrated VSV transmission via mosquitoes [32,34,50,51]. Although many experts are skeptical of mosquitoes’ ability to transmit VSV due to their feeding habits, their role in VSV transmission has not been ruled out in either epizootic or enzootic settings [32,33,52,53,54].

Studies of the vectors involved in VSV transmission in its enzootic range are much scarcer than those in regions where it causes outbreaks. VSV has only been isolated from four (of sixty-eight) pools of field-collected *Lutzomyia* spp. sand flies from Panama [30,55], one (total number of pools screened not reported) pool of *Culex nigripalpus* mosquitoes from Guatemala, and one (total number of pools screened not reported) pool of *Mansonia indubitans* mosquitoes from Ecuador [54] (Figure S1). To begin to fill this knowledge gap and draw inference about the determinants of VSV endemicity, we conducted a longitudinal study of VSV transmission dynamics between July 2021 and December 2022 in five cattle ranches within the endemic region of the virus in Chiapas, Mexico. At each ranch, we monitored livestock VSV cases using passive surveys, screened serum samples for anti-VSV neutralizing antibodies, sampled hematophagous insects in different land use and land cover (LULC) types, and screened these insects for VSNJV and VSIV. To infer the transmission dynamics of VSV, we compared the number of VS cases reported by season with the relative abundance of, and VSV prevalence in, the four sampled insect taxa across the three major seasons of Chiapas: rainy, post-rainy, and dry. Additionally, we compared these variables and the seroprevalence of VSNJV to VSIV to gain insight into the similarity, or lack thereof, in the patterns of transmission of the two viruses.

## 2. Materials and Methods

### 2.1. Sample Site Selection

**Figure 1:**
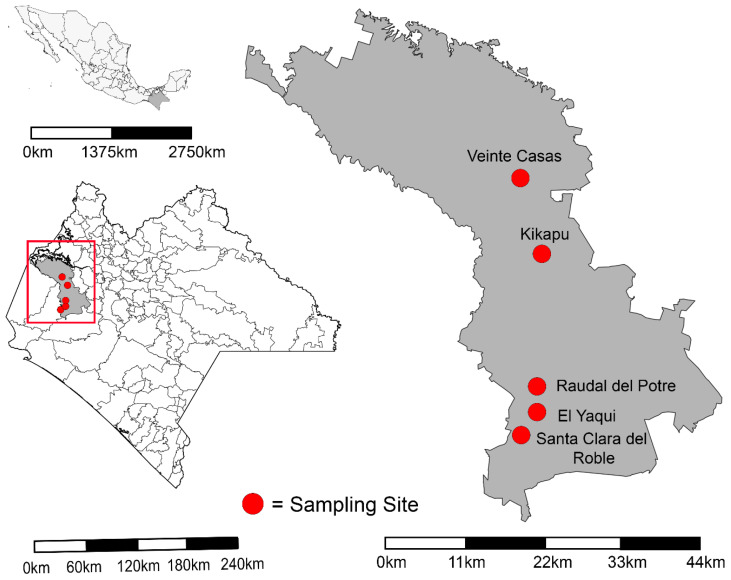
Map of Chiapas State in Mexico and the municipality of Ocozocoautla de Espinosa (red box). Sampled ranches and names are indicated by red dots.

**Figure 2:**
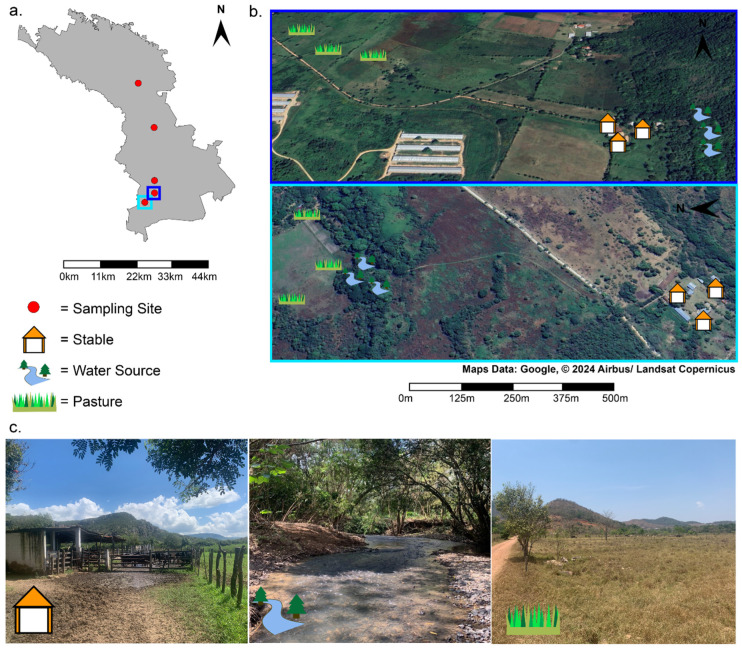
Sampling sites and land use and land cover (LULC) types. (**a**) Map of Ocozocoautla de Espinosa and sampled ranches showing the locations of two representative ranches. (**b**) Ranch Santa Clara del Roble (**top**; blue) and Ranch El Yaqui (**bottom**; light blue), with trap locations shown. (**c**) Representative photos for each LULC type are included. Images of all ranches are shown in Supplementary Figures S2 and S3.

Five cattle ranches in Chiapas, Mexico, with a history of VS cases reported by ranch owners were selected for this study. For the safety of project personnel, all ranches were located within the municipality of Ocozocoautla de Espinosa, Chiapas, Mexico (Figure 1 and Table S1). Consent and permission to conduct insect and serum collections were obtained at each ranch. All ranches held bovine and equine species, while sheep, pigs, and goats were present at some ranches. At each ranch, three general LULC types were delineated (Stable, Water Source, Pasture) as follows: Stable—pens and enclosures housing cattle and horses. Mostly dirt and low vegetation substrate; Water source—bodies of water (seasonal, perennial) found on ranch property, usually a stream or small pond, that provide drinking water for livestock; and Pasture—grass-covered lands where cattle regularly graze (Figure 2, Figures S2 and S3). Within each of the three LULC types, three sampling sites were selected for insect trap placement to maximize the separation and representation of environmental variability as visually estimated by the investigators. This resulted in a total of nine sampling sites per ranch. Due to the varying configurations of ranches and LULC types, sampling sites within the same LULC type were between 30 and 150 m apart (Figure 2, Figures S2 and S3).

### 2.2. VS Clinical Case Data Collection

Clinical livestock cases were passively monitored at our sampling sites via surveys (Figure S4) handed out to the head of each ranch at the start of each sampling season and collected at the end of each season. Surveys captured metadata for each case, including case species, age, sex, reproductive state, whether the animal was acquired from a different property, symptoms, and date of infection.

### 2.3. Serosurveillance

Between 21 November and 21 December 2021, cattle sera were collected by veterinarians of the Comisión México-Estados Unidos para la Prevención de la Fiebre Aftosa y otras Enfermedades Exóticas (CPA) as part of their routine serosurveillance for VSV in the area. Sera were collected from 32 cows (16 adults (>12 months of age) and 16 juveniles (≤12 months of age)). If a ranch did not have 16 juveniles, sera were sampled from additional adult cattle to obtain sera from 32 individuals per ranch (Raudal del Potro, Veinte Casas, and Santa Clara del Roble). Serum samples were screened via neutralization assays, described below. Age was also recorded for all animals at each ranch except one, where animals were only identified as adult or juvenile (Ranch Kikapu). Blood samples were collected from the jugular or coccygeal vein from every individual using a 21 G × 38 mm needle and transferred into vacutainer serum tubes with clot activator gel (BD, Franklin Lakes, NJ, USA) by official veterinarians of the CPA. After collection, samples were stored at ambient temperature for 15 min, centrifuged at 1000× *g* for 10 min, transferred into 1.8 mL sterile vials, and stored at −80 °C.

Neutralizing antibodies against VSNJV and VSIV in sera were detected using a previously described neutralization assay [56]. Briefly, collected serum samples were heat-inactivated at 56 °C for 30 min. Next, serial dilutions of heat-inactivated sera were incubated with 1000 TCID_50_ of VSNJV or VSIV (NJ0612NME6, IN0919WYB2, respectively, Plum Island Animal Disease Research Center) for 1 h at 37 °C in 96-well plates. Following incubation, 100 µL of serum/virus mixture were added to pre-plated 96-well tissue culture-treated plates (Fisher Scientific, Waltham, MS, USA) containing Vero cell (Vero CCL-81, ATCC, Manassas, VA, USA) monolayers and incubated for 3 days at 37 °C with 5% CO_2_. Vero culture medium consisted of EMEM (ATCC, Manassas, VA, USA) supplemented with 10% FBS and 1× antibiotic–antimycotic (Fisher Scientific, Waltham, MS, USA). Cytopathic effect was scored, and serum neutralizing activity was reported as the reciprocal of the highest dilution giving 100% inhibition of CPE. A 1:32 titer was established as the cutoff point for seropositivity in accordance with the World Organization of Animal Health Manual of Diagnostic Tests and Vaccines for Terrestrial Animals [57].

### 2.4. Insect Sampling

To delineate sampling seasons, 2020 precipitation data (retrieved from Daymet.org, accessed 4 April 2021) were used to separate the year into three seasons: dry season (January–April), rainy season (May–September), and post-rainy season (October–December). At each ranch, insect sampling occurred once per season for three consecutive days. In 2021, insect sampling occurred only during the rainy and post-rainy seasons. In 2022, insect sampling occurred during all three seasons. The specific dates of the sampling seasons were: 2021 rainy season (5 July–6 August 2021), 2021 post-rainy season (1 November–3 December 2021), 2022 dry season (15 March–14 April 2022), 2022 rainy season (15 August–16 September 2022), and 2022 post-rainy season (17 October–16 November 2022). During each sampling season, US Centers for Disease Control (CDC) miniature light traps baited with dry ice (light source removed) were placed at each LULC type as described above. Traps were hung on trees or fences, approximately 1–1.5 m from the ground. Traps were placed after 10:00 h and no later than 16:00 h on the first day of sampling. Dry ice, batteries, and collection nets were then replaced daily between 10:00 and 14:00 h until the final (third) day of sampling. Traps were collected between 10:00 and 14:00 h on the final (fourth) day. All collections nets were transported back from the field in a cooler with ice packs and placed in a −80 °C freezer each day.

### 2.5. Insect Sorting, Pooling, and Morphological Identification

Insects were identified morphologically on a Petri dish placed atop frozen ice packs, counted, and pooled by taxon/morphotype, site, LULC type, trap and date before being returned to −80 °C to maintain the cold chain. Due to the lack of comprehensive taxonomic keys for the region, insects were first morphologically identified by genera or family. From there, morphotypes corresponding to putatively different species were then identified, and insects were pooled by morphotype in pools of 20 or fewer individual insects for downstream molecular analyses. Psychodidae were identified based on morphological characters presented in previously published guides [58,59], mainly in the wings and thorax. Culicidae were identified based on morphological characters presented in Carpenter and LaCasse [60]. Ceratopogonidae were identified based on wing morphology [61,62,63], and Simuliidae were also identified based on morphological characters like body color, leg color, and size [64,65]. Abundance was recorded, and samples were returned to −80 °C for downstream molecular analyses.

### 2.6. RNA Isolation and VSV RNA Detection in Insects

Because it was not possible to screen every pool of the four putative vector taxa (Simuliidae, Psychodidae, Ceratopogonidae, and Culicidae) due to resource limitations, a subset of 874 pools was selected from the first three sampling seasons (2021 rainy season, 2021 post-rainy season, and 2022 dry season) to screen for VSIV and VSNJV RNA. For each sampling season, 25 pools from each of the four putative vector taxa were selected from each ranch. If a taxon had fewer than 25 pools available from a ranch, all available pools for that taxon were chosen. When more than 25 pools were available, 25 pools were randomly selected across all LULC types by assigning each pool a number and using a random number generator. To extract RNA, pools were homogenized using a motorized tissue grinder and pestle for five minutes and in 500 µL of phosphate-buffered saline (PBS). Trizol LS reagent (Invitrogen, Waltham, MS, USA) was used to extract total RNA from the samples, following the manufacturer’s instructions. Isopropanol was used to precipitate RNA from the first aqueous phase before two subsequent washes with 75% ethanol. The final RNA product was resuspended in 50 µL of nuclease-free water. Extracted total RNA samples were screened for VSNJV and VSIV RNA using the CFIA-NCFAD multiplex qRT-PCR protocol [66] targeting the VSV L gene with an ABI 7500 system (Applied Biosystems, Austin, TX, USA). For each sample, a 25 µL amplification reaction consisting of 5 µL of RNA template, 6.25 µL of 4× Taqman Fast Virus 1-step Mastermix (Applied Biosystems, Waltham, MA, USA), 12.25 µL of RNAse-free water, and a 1.5 µL cocktail of VSIV and VSNJV forward primers (0.2 µM each), reverse primers (0.2 µM/0.8 µM, respectively), and probes (0.1 µM, 0.05 µM, 0.05 µM, 0.1 µM, respectively) (Table S2), was subjected to the following cycling conditions: 50 °C for 5 min, 95 °C for 20 s; and 40 cycles of 95 °C for 15 s and 60 °C for 45 s. All PCR reactions included a positive control for each serotype provided by the BSL3 national diagnostics laboratory of the CPA (Palo Alto, Ciudad de Mexico); RT-qPCR reactions with a Ct < 35 were considered positive for VSV [66,67,68].

### 2.7. Virus Isolation

Macerated insect samples from five qRT-PCR-positive pools were filtered via centrifugation through 0.45 mm Spin-X filter columns (Costar cat. No 8163). The cleared fluids were diluted 1:2 in maintenance medium consisting of EMEM (ATCC, Manassas, VA, USA) supplemented with 2% FBS and 1× antibiotic–antimycotic (Fisher Scientific, Waltham, MA, USA), and 500 µL of each dilution was applied to Vero cell (CCL-81, ATCC, Manassas, VA, USA) monolayers in 24-well plates and monitored for cytopathic effect (CPE) for 72 h.

### 2.8. Cytochrome C Oxidase Subunit I (COI) Barcoding

To bolster morphological identifications of target taxa, COI barcoding was conducted on 138 insect pools. Of the 874 pools screened for VSV RNA, DNA was extracted from all pools that tested positive for VSV RNA (*n* = 101), excluding pools used for viral isolation. Additional samples that tested negative for VSV RNA (*n* = 32) were also included to expand taxonomic diversity. DNA was extracted from macerated insect samples using Zymo Quick-DNA Tissue/Insect kits (Zymo Research, Irvine, CA, USA) following the manufacturer’s instructions; DNA was eluted in 25 µL of elution buffer from the kit.

Following a previously described COI barcoding protocol [43], COI amplicons were generated from 100 ng sample DNA and universal COI primers designed by Folmer et al. [69] (Table S2). The PCR cycling conditions were 96 °C for 1 min; 35 cycles of 94 °C for 1-min, 52 °C for 1 min; 72 °C for 1.5 min; and 8 °C once completed. Amplicons were detected via gel electrophoresis with 10 µL of PCR reaction. If an amplicon was visible, 15 µL of PCR product was purified using the Roche High Pure PCR Product Purification kit (Sigma-Aldrich, St. Louis, MO, USA), following the manufacturer’s instructions, and subjected to Sanger sequencing. Chromatograms were analyzed and cleaned in Geneious Prime version 2021.1.1 (https://www.geneious.com (accessed on 20 March 2024)). Sequences with low base-call quality scores (<90%) were removed from downstream analyses. Forward and reverse reads were aligned using the MAFFT aligner [70] to generate consensus barcode amplicons, and sequences less than 550 bp in length were excluded from downstream analyses.

### 2.9. Analysis

Phylogenetic trees were constructed with generated barcode amplicons and published GenBank sequences from all Culicidae, Psychodidae, and Ceratopogonidae species documented by recent studies in Mexico [71,72,73]; Simuliidae were scarce in our overall collection and yielded no barcodes. To obtain reference sequences for species documented in Mexico, we searched NCBI GenBank for available COI barcode sequences using species-level scientific names. When multiple search results were found, a single sequence was chosen based on the following criteria: Sequences generated from samples collected in Mexico were prioritized. If not available, sequences from North or South America were selected, with a preference given to countries geographically closer to Southern Mexico. In two cases (*Culex pipiens* and *Culicoides pusillus*), sequences from Bangladesh and France were used. All reference GenBank sequences used in phylogenetic analyses are listed in Table S3.

For each family, generated barcodes and GenBank reference sequences were aligned using the MAFFT aligner [70] and manually trimmed. The *Simulium meridionale* COI barcode sequence (GenBank Accession: OR459455.1) was used as an outgroup for all phylogenies. Maximum likelihood phylogenies were reconstructed for each family using RAxML Version 8 [74] using the GTR evolutionary model and 1000 bootstrap (BS) replicates. For Culicidae, individual genus-level phylogenies were constructed for *Aedes*, *Culex*, and *Psorophora*. A phylogeny consisting of multiple genera (*Wyeomyia*, *Sabethes*, *Coquillettidia*, and *Mansonia*) was also constructed based on recently published phylogenetic Culicidae mitogenomic relationships [75]. Because Culicidae (*Aedes* spp. and *Culex* spp.) and Ceratopogonidae (*Culicoides* spp.) phylogenies constructed with single GenBank reference sequences contained unresolved clades, additional GenBank sequences were added to obtain better resolution for these clades.

### 2.10. Statistical Analyses

For serosurveillance data, a contingency table analysis comparing livestock seropositivity data among ranches was conducted to assess whether the proportion of seropositive animals differed. A Mann–Whitney U Test comparing VSNJV neutralizing antibody titers between juvenile and adult cattle (pooled across ranches) was also conducted. For insect abundance, a two-way analysis of variance (ANOVA) test was conducted to determine whether abundance differed among taxa (family), sampling season, or their interaction. The distribution of insect abundance data was heavily left-skewed, so the data were transformed as log_10_ + 1 per data point. If a statistically significant effect was detected from the two-way ANOVA, Tukey’s honestly significant difference (HSD) post hoc test was implemented. To investigate whether insect abundance (irrespective of taxa) differed among LULC types by season, we conducted one-way ANOVA tests using log_10_ +1-transformed insect abundance and LULC for each season. For virus screening data, a nominal logistic regression was conducted to investigate whether the number of VSNJV-positive samples differed among taxa (family), LULC, sampling season, or their interactions (sampling season*LULC and sampling season*taxa). We conducted post hoc contingency table analyses and Fisher’s exact tests to identify specific pairwise differences among groups. All analyses were run in JMP version 17.0 (SAS Institute Inc., Cary, NC, USA).

## 3. Results

### 3.1. Livestock VS Cases Reported During Rainy and Post-Rainy Seasons

**Figure 3:**
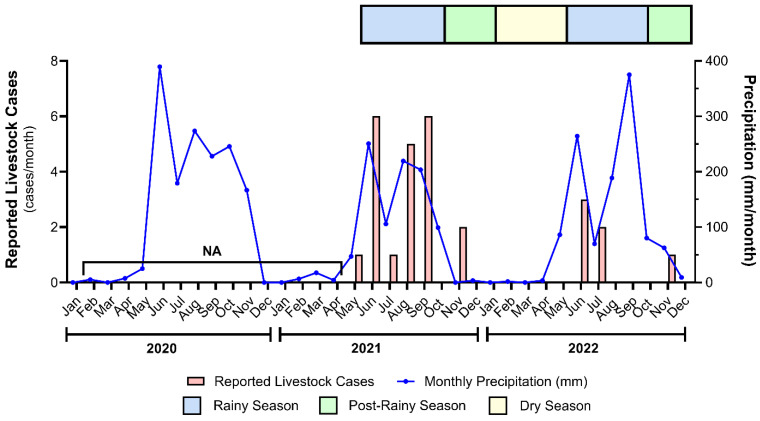
The 2020–2022 monthly precipitation from Ranch Santa Clara del Roble, sampling season designations, and reported vesicular stomatitis (VS) livestock cases from the five ranches. Surveys were not distributed from January 2020 to April 2021 (“NA”).

Livestock VS cases were captured by survey from May 2021 to December 2022 (Figure 3 and Table S4). All clinical cases reported were cattle, and cases were reported from every ranch except for Ranch Veinte Casas. Clinical cases were also reported by ranches in both years. Livestock clinical cases were highest during the rainy season, and no cases were reported during the one dry season sampled (January to May 2022) (Figure 3 and Table S4). Infected individuals ranged from 15 days to 11 years old, and one re-infection case was documented (Regalo, 6-year-old female cow from Ranch Raudal del Potro, infected 30 June 2021 and 10 November 2021). One VSV lesion was collected from an infected cow from Ranch Santa Clara del Roble on 21 November 2021; VSNJV was isolated, and the coding-complete genome sequence has been deposited in GenBank under accession no. PQ181559.

### 3.2. High VSNJV Seroprevalence in Cattle

**Figure 4:**
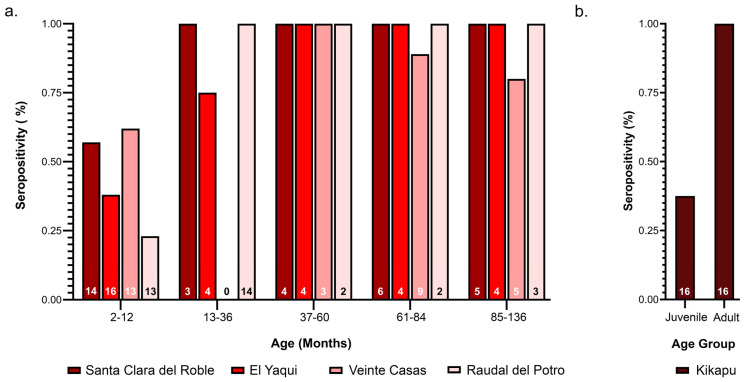
Percentage of cattle of designated age that were seropositive for Vesicular stomatitis New Jersey virus (VSNJV) per ranch; N at each ranch is listed within each bar. (**a**) VSNJV seropositivity at ranches that provided specific age data; (**b**) VSNJV seropositivity at Rancho Kikapu, which only provided age class data.

The majority of cattle at the five ranches were seropositive for VSNJV, with both juvenile and adult animals displaying high seropositivity. For individuals younger than one year, seropositivity ranged from 20 to 65% per ranch, while seropositivity in adults ranged from 75 to 100% per ranch (Figure 4 and Table S5). Across all ages, 72% of all animals tested were seropositive for VSNJV. Neutralizing antibody titers in seropositive individuals ranged from 1:25,000 to 1:32, and high antibody titers were observed across all ages. A significant difference in VSNJV neutralizing antibody titers was detected between adult and juvenile cattle (S = 4030.5, Z = −7.27, *p* < 0.0001). Of all animals sampled, only one individual tested seropositive for VSIV (1:40). No differences were detected in the relative frequency of VSNJV seropositive individuals between ranches (contingency table analysis, df = 4, χ^2^ = 2.32, *p* = 0.67).

### 3.3. All Four Taxa of Implicated Vectors Present on All Five Ranches in All Three Sampling Seasons

**Figure 5:**
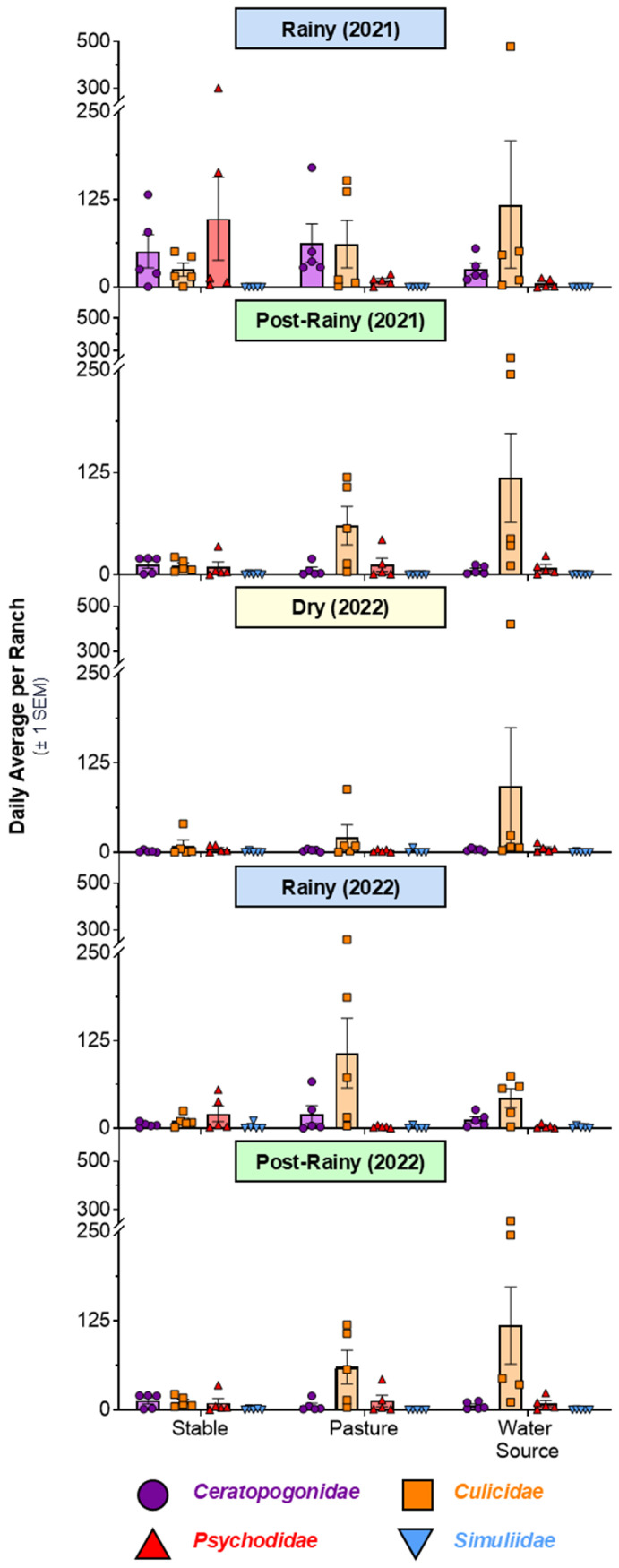
Daily average (±standard error of the mean (SEM)) number of designated insect taxa sampled per ranch per sampling season. Note that outlier *Psorophora* spp. counts have been removed from the 2021 rainy season (see Supplementary Figure S5). Results are colored by insect taxa and grouped by LULC type and season. Colored bars represent the average of 5 sampled ranches.

In each sampling season, individuals from all four potential vector taxa were collected in all three seasons, including Ceratopogonidae (*n* = 3007), Culicidae (16,977), Psychodidae (4805), and Simuliidae (450) (Figure 5). The total number of insects sampled per season, taxon, ranch, and LULC types are provided in Supplementary File S1.

During the first year of sampling, a large *Psorophora* spp. (Culicidae) population boom occurred during the 2021 rainy season in one ranch (Ranch Kikapu; N > 5500 individuals). This was likely due to the annual flooding reported at this ranch; similar population explosions attributable to flooding have been documented in *Psorophora* species in the USA [76]. As a result, *Psorophora* spp. data from this ranch and season were removed from statistical analyses (Figure 5 and Figure S5) but were still included in VSV qRT-PCR screening. Due to the very low number of Simuliidae samples collected, Simuliidae species were removed from statistical analyses but were also included in VSV qRT-PCR screening.

The two-way ANOVA test revealed a significant interaction effect of taxa (family) and sampling season on abundance (F_(8,210)_ = 3.95, *p* = 0.0002). Ceratopogonidae abundance was significantly higher during the 2021 rainy season compared with the 2021 post-rainy season, as well as the 2022 dry and post-rainy seasons. No differences were detected in Culicidae abundance by season. Psychodidae abundance was higher in the 2021 rainy season compared with the 2022 rainy and post-rainy seasons (Table 1). One-way ANOVA tests by season did not reveal significant effects for LULC type on insect abundance (*p* > 0.11 for all tests).

### 3.4. VSNJV Detected in All Four Implicated Vector Taxa in All Three Sampling Seasons; VSIV Detected Only in Culicidae in the Rainy Season

**Figure 6:**
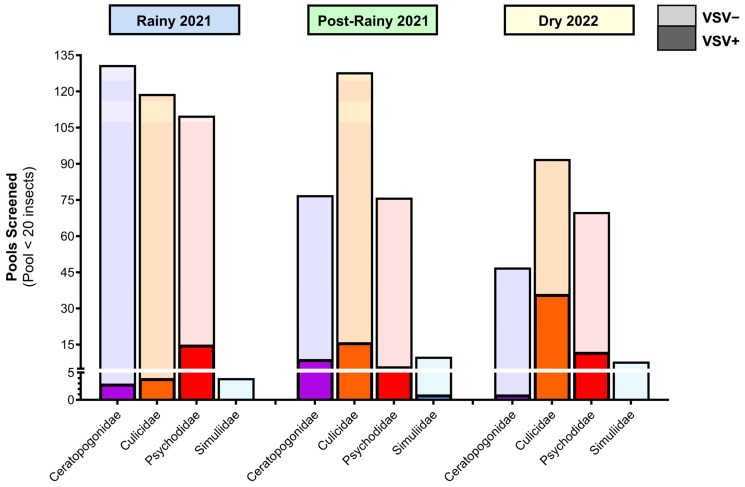
Insect VSNJV screening results by season (qRT-PCR). Dark shaded regions represent VSNJV-positive pools, and light shaded regions depict VSNJV-negative pools.

**Figure 7:**
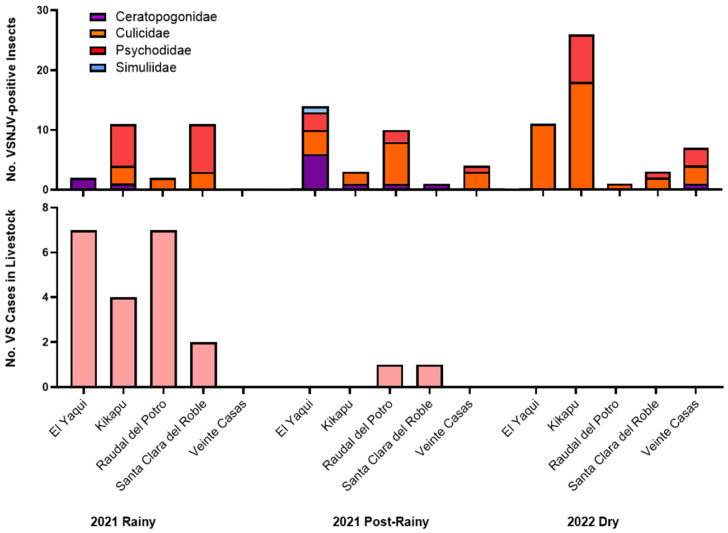
Total number of VSNJV-positive insects (**top**) and reported VS cases in livestock (**bottom**) by ranch and sampling season.

In total, 874 pools (6186 insects total) were selected from the first three sampling seasons (2021 rainy season, 2021 post-rainy season, 2022 dry season) for VSIV and VSNJV RNA screening via qRT-PCR. VSNJV RNA was detected in 102 pools of insects from all four taxa of interest and from all five ranches (Figure 6). VSIV RNA was only detected in four pools of Culicidae during the 2021 rainy season at Ranches Santa Clara del Roble and Kikapu (one pool morphologically identified as *Psorophora* spp.; the other three pools were not identified at the genus level). Of 48 Simuliidae individuals screened, one pool from Ranch El Yaqui tested positive for VSNJV RNA during the post-rainy season. Culicidae, Psychodidae, and Ceratopogonidae pools from all three seasons also tested positive for VSNJV RNA (Figure 6). VSV RNA was detected in insects during the dry season, but no livestock cases were reported during that season (Figure 7). Complete insect qRT-PCR screening results can be found in Supplementary File S2. Viral isolation was attempted using five Culicidae pools that tested positive for VSV (four VSIV-positive pools and one VSNJV-positive pool) but was unsuccessful.

A nominal logistic regression analysis identified significant interaction effects between both sampling season and LULC (χ^2^ = 15.65, *p* = 0.003), as well as sampling season and taxa (χ^2^ = 21.18, *p* = 0.0003). During the rainy season, the number of VSV-positive insects were higher in “Stable” sites compared with “Water Source” sites (one-tailed, *p* = 0.007), but no differences were detected when comparing these sites with “Pasture” sites (two-tailed, *p* > 0.15 for all tests). During the post-rainy season, the number of VSV-positive insects were higher in both “Stable” and “Pasture” sites when compared with “Water Source” sites (one-tailed, *p* = 0.005 and *p* < 0.0001, respectively). No differences were observed between “Stable” and “Pasture” sites during the post-rainy season (two-tailed, *p* = 0.20). During the dry season, no differences were observed in VSV-positive insects between LULC types (two-tailed, *p* > 0.53 for all tests). Regarding sampling season and taxa, a higher number of VSV-positive Culicidae and Psychodidae were detected during the rainy season compared with Ceratopogonidae (one-tailed, *p* = 0.03 and *p* < 0.0001, respectively). In addition, the number of VSV-positive Psychodidae were also higher than VSV-positive Culicidae (one-tailed, *p* = 0.04). During the post-rainy season, no significant differences were detected in VSV-positive insects by taxa (two-tailed, *p* > 0.58 for all tests). During the dry season, the number of VSV-positive Culicidae were significantly higher than both Psychodidae and Ceratopogonidae (one-tailed, *p* = 0.0006 and *p* < 0.0001, respectively). The number of VSV-positive Psychodidae were also higher than Ceratopogonidae in the dry season (one-tailed, *p* = 0.007).

### 3.5. COI Barcodes for VSV-Positive Ceratopogonidae, Psychodidae, and Culicidae

**Figure 8:**
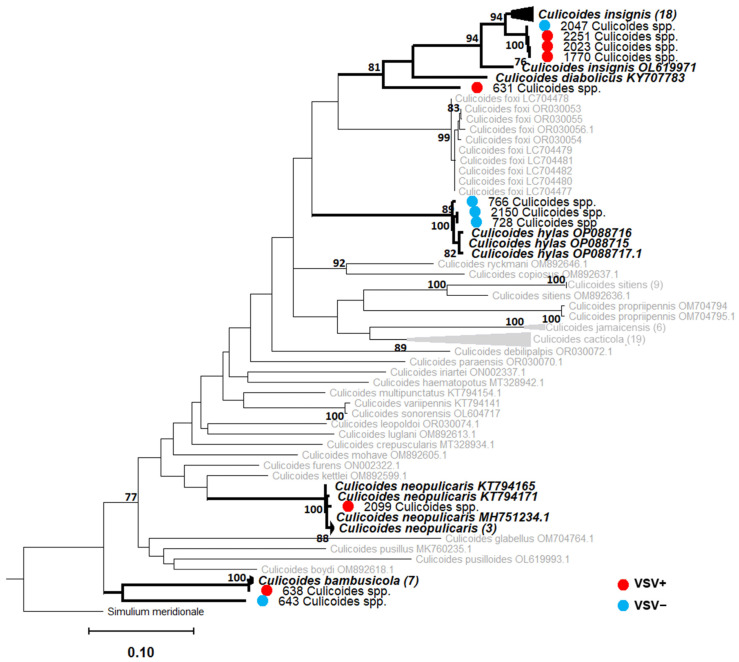
Ceratopogonidae (*Culicoides* spp.) maximum likelihood (ML) phylogeny RAxML v.8; bootstrap values >75% (1000 replicates) are noted at the nodes. For the generated barcodes, screening results are indicated with a red (VSNJV-positive) or blue (VSNJV-negative) circle, followed by the sample ID and morphological identification. GenBank sequences are displayed with accession numbers. Clades containing both generated barcodes and reference GenBank sequences are in bold formatting. The remaining GenBank sequences not placed within the same clade as our barcodes are in gray. The number of sequences contained in collapsed nodes are listed in parentheses.

**Figure 9:**
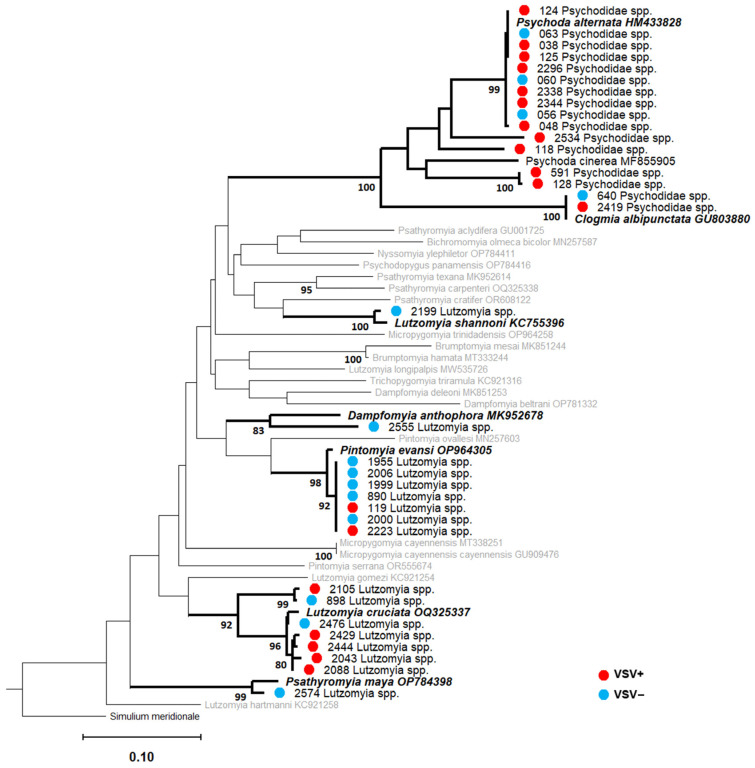
Psychodidae ML phylogeny; RAxML v.8; bootstrap values >75% (1000 replicates) are noted at the nodes. For generated barcodes, screening results are indicated with a red (VSNJV-positive) or blue (VSNJV-negative) circle, followed by the sample ID and morphological identification. GenBank sequences are displayed with accession numbers. Clades containing both generated barcodes and reference GenBank sequences are in bold formatting. The remaining GenBank sequences not placed within the same clade as our barcodes are in gray. The number of sequences contained in collapsed nodes are listed in parentheses.

**Figure 10:**
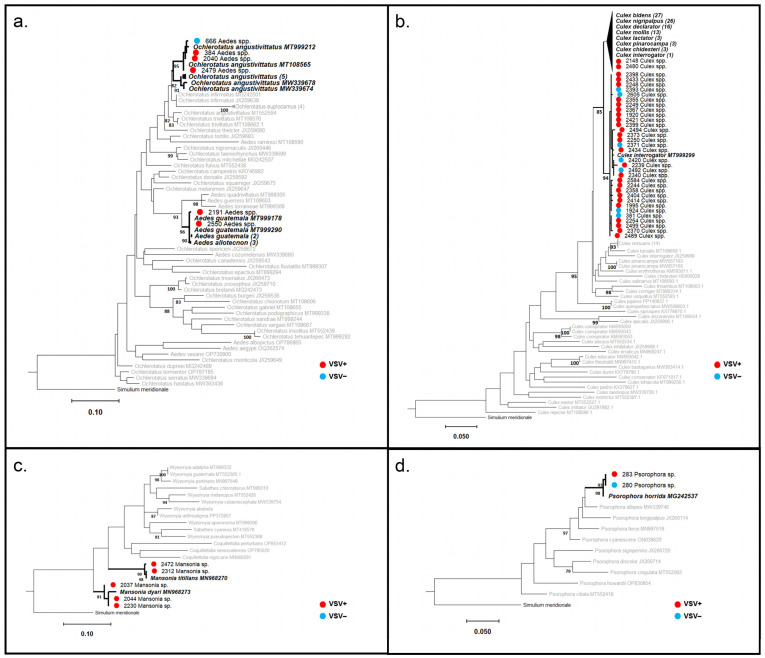
Culicidae ML Phylogenies; RAxML v.8; bootstrap values >75% (1000 replicates) are noted at the nodes. For generated barcodes, screening results are indicated with a red (VSNJV-positive) or blue (VSNJV-negative) circle, followed by the sample ID and morphological identification. GenBank sequences are displayed with accession numbers. Clades containing both generated barcodes and reference GenBank sequences are in bold formatting. The remaining GenBank sequences not placed within the same clade as our barcodes are in gray. The number of sequences contained in collapsed nodes are listed in parentheses. (**a**) *Aedes* spp. phylogeny; (**b**) *Culex* spp. phylogeny; (**c**) *Mansonia* spp. phylogeny (with *Wyeomyia*, *Haemagogus*, and *Coquillettidia* spp.); (**d**) *Psorophora* spp. phylogeny.

We generated clean COI amplicons for 91/138 pools (64 VSNJV-positive pools, 27 VSNJV-negative pools). Of those 91 pools, 51 pools were 97–99% identical to a reference sequence from NCBI GenBank and were phylogenetically positioned within the same clade as their reference sequences with >75% bootstrap support (Figure 8, Figure 9 and Figure 10 and Figures S6–S8 and Table S6). As a result, we were able to confidently assign 51 species-level identifications to 31 VSNJV-positive pools and 20 VSNJV-negative pools. For the remaining 40 generated barcodes without species-level phylogenetic resolution (bootstrap < 75), genus-level identifications (with spp.) were assigned based on BLAST searches and phylogenetic reconstruction for all samples except for pool 643 (which aligned with Ceratopogonidae sp. sequence with 88% identity; GenBank accession number: OM602255.1). Of the 39 sequences limited to genus-level identifications, 34 belonged to the genus *Culex* spp. (Culicidae), 4 to *Psychoda* spp. (Psychodidae), and 1 to *Culicoides* sp. (Ceratopogonidae) (Figure 8, Figure 9 and Figure 10 and Figures S6–S8).

**Figure 11:**
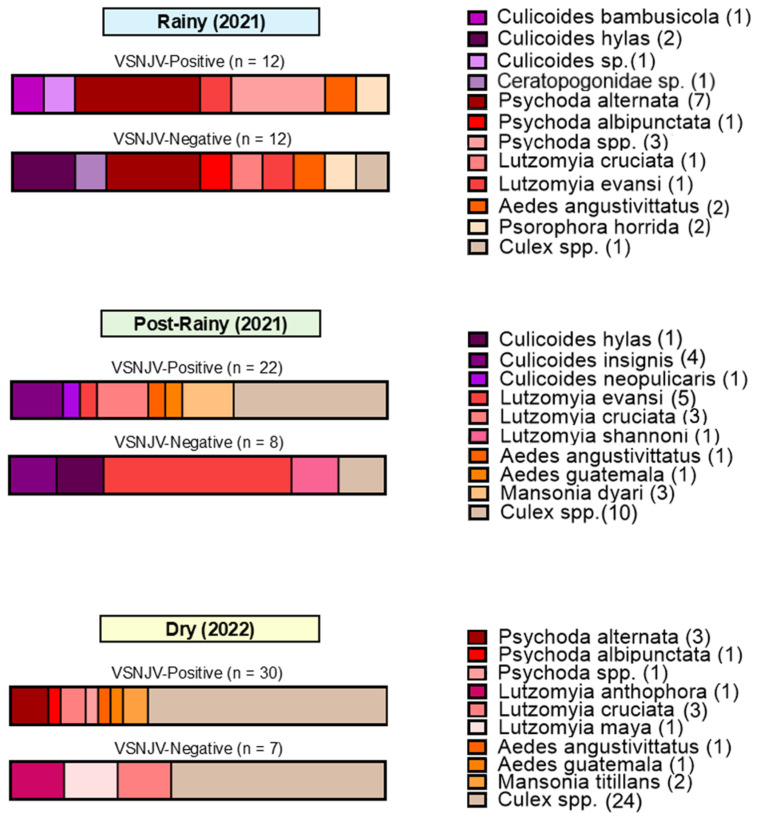
Species identified by the DNA barcoding of VSNJV-positive and VSNJV-negative pools by taxon and sampling season. The total number of each species per sampling season are listed in parentheses.

Based on these identifications, twelve different species from three families tested positive for VSNJV RNA, including three Ceratopogonidae species (*Culicoides bambusicola*, *Culicoides insignis*, and *Culicoides neopulicaris*), four Psychodidae species (*Lutzomyia* (*Pintomyia*) *evansi*, *Lutzomyia cruciata*, *Psychoda alternata*, and *Psychoda* (*Clogmia*) *albipunctata*), and five Culicidae species (*Aedes* (*Ochlerotatus*) *angustivittatus*, *Aedes guatemala*, *Psorophora horrida*, *Mansonia dyari*, and *Mansonia titillans*) (Figure 8, Figure 9 and Figure 10 and Figures S6–S8). Of the 39 pools limited to genus-level identifications, VSNJV RNA was detected in 28 pools of *Culex* spp., 4 pools of *Psychoda* spp., and 1 pool of *Culicoides* sp. *1* (Figure 8, Figure 9 and Figure 10 and Figures S6–S8). For the rainy and post-rainy seasons, barcodes were generated from VSNJV-positive Ceratopogonidae, Culicidae, and Psychodidae, while only Culicidae and Psychodidae barcodes were generated for the dry season (Figure 11). Small sample sizes prevented statistical analysis, but the species with barcodes that only yielded VSNJV-positive results were *Culicoides neopulicaris* (*n* = 1), *Culicoides bambusicola* (*n* = 1), *Culicoides* sp. (*n* = 1), *Aedes guatemala* (*n* = 2), *Mansonia dyari* (*n* = 3), *Mansonia titillans* (*n* = 2), and *Psychoda* spp. (*n* = 4). In contrast, a few species were only VSNJV-negative, including *Lutzomyia shannoni* (*n* = 1), *Lutzomyia* (*Psathyromia*) *maya* (*n* = 1), *Lutzomyia* (*Dampfomyia*) *anthophora* (*n* = 1), and *Ceratopogonidae* sp. (*n* = 1). The remaining barcoded species tested both VSNJV-positive and VSNJV-negative (*Lutzomyia evansi*, *Psychoda alternata*, *Psychoda albipunctata*, *Aedes* (*Ochlerotatus*) *angustivittatus*, *Culex* spp., and *Psorophora horrida*) (Figure 11).

## 4. Discussion

To gain insight into the environmental and biological factors that promote endemicity of VSV, we investigated the occurrence of VSV in livestock and implicated arthropod vector taxa on five ranches in Chiapas, Mexico. First, we sought to define the seasonality of case occurrence and the dynamics of infection using case reports in the three major seasons in Chiapas (dry, rainy and post-rainy). Livestock cases were reported at four of the five ranches, but only during the rainy and post-rainy seasons. Similarly, Mexico’s National Epidemiological Surveillance System (SIVE) reported VS cases in Chiapas from July 2021 (the 2021 rainy season) continuously through November 2021 (the 2021 post-rainy season), but cases were not reported between December 2021 and June 2022 (the 2022 dry season). The concentration of VS cases in the rainy and post-rainy seasons has also been reported in previous studies in Southern Mexico [12,77]. VSV was isolated and sequenced from a lesion from one of the cases from our ranches, revealing a VSNJV strain that was genetically similar to others previously sequenced from cattle in the region. We also detected a symptomatic re-infection in one cow, with a first infection in June 2021 and a second infection in November 2021. This can be explained by prior reports that the presence of neutralizing antibodies in cattle is not enough to prevent clinical disease, as many animals have tested positive for antibodies prior to infection in endemic areas [2,11,78,79].

Next, we conducted serosurveillance to draw inference about the force of VSV infection. Our serosurvey showed extremely high levels of VSNJV seroprevalence in cattle tested from all five ranches, nearing 100% of sampled animals older than one year, indicating an extremely high force of infection. As expected, seropositivity increased with age, but even juvenile animals showed high seroprevalence, some of which may be attributable to the persistence of maternal antibodies [2]. The higher seroprevalence of VSNJV compared with VSIV is also consistent with our current knowledge of VSV epidemiology. The VSNJV serotype is responsible for over 80% of clinical cases reported in the United States and 90% of yearly VS cases reported in the endemic Mexican states of Veracruz, Chiapas, and Tabasco [7,13,14]. Additionally, virus neutralization assays from outbreaks of VS in Mexico between 1981 and 2012 show that more than 60% of clinical infections correspond to VSNJV [12]. Similar levels of VSNJV seroprevalence in other VSV endemic regions such as Costa Rica are also consistent with our results [11,77,78,80,81].

Additionally, we collected hematophagous insects from all four of the taxa implicated as potential VSV vectors (black flies, sandflies, midges, and mosquitoes) to compare vector identity, diversity, abundance across seasons, and among different LULC types. All four taxa were present in all five ranches in all three sampling seasons, but black flies were extremely scarce. The abundance of particular taxa waxed and waned across seasons, and a decline in Ceratopogonidae and Psychodidae abundance was also observed in the dry season. No statistically significant differences were observed in insect abundance across the different LULC types.

We screened a subset of collected insects for VSV RNA to compare viral dynamics in insects across seasons, taxa, and LULC types. VSNJV was detected in 11% of insect pools screened (102/874), including all four taxa on all five ranches. VSIV was only detected from a small subset of pools (4/874), all of which were mosquitoes, and only in the rainy season. An important caveat is that the detection of VSV RNA in an insect via qRT-PCR does not demonstrate the insect as a competent VSV vector, as VSV RNA can be detected from bloodmeals that do not seed forward-going transmission or from surface contamination of the insect. As a result, the detection of VSV RNA in non-hematophagous *Psychoda* spp. flies in this study is not surprising. We suspect these pools acquired the virus mechanically, as has been documented in grasshoppers, houseflies, and other non-hematophagous flies [41,82]. Despite this, the detection of VSV RNA in a wide variety of insect species indicates the presence of VSV at these ranches and suggests that multiple insect species may be involved in VSV transmission. Furthermore, the differences between VSIV and VSNJV in both seroprevalence in livestock and occurrence across time and taxa in insects may indicate distinct transmission cycles of the two viruses.

VSNJV was detected in at least a subset of taxa in all seasons, and the prevalence was relatively similar across all three seasons. These observations support the hypothesis that Chiapas represents a “Goldilocks zone” (referencing the fairy tale in which the perfect porridge for a little girl was neither too hot nor too cold), enabling year-round transmission of VSV. In the epizootic region of the USA, in contrast, VSV livestock cases cease in response to major environmental changes that suppress insect activity, such as hard winter frost or the damming of a major waterway [1,2,43,83].

The detection of VSNJV RNA-positive insects during the dry season, when no cases were detected via our surveys or by SIVE, was unexpected. Although subclinical VSV infection of livestock is common, viral transmission from infected hosts to insect vectors is thought to occur only when vectors feed on lesions, as viremia is undetectable in infected livestock [35,36,37,84,85]. Multiple, non-exclusive hypotheses may explain this disconnect. First, if reporting is inadequate, low numbers of cases in the dry season may be missed. We find this unlikely for our ranches, where we communicated regularly with each rancher about case occurrence and cattle were closely monitored by them for VSV lesions. Second, individual insects infected in the post-rainy season may survive through the dry season. This is also unlikely, given that the reported lifespans of these insects range from 36 to 100 days on average, but longer lifespans have been recorded for mosquitoes [86,87,88]. In contrast, dry seasons in Chiapas can last up to 4–5 months, which is longer than the average recorded lifespans of potential insect vectors. Third, insects may transmit the virus among themselves without need of an infected host through transovarial or venereal transmission or through co-feeding [89]. Orally infected sandflies (*L. shannoni*) were able to pass VSNJV to 23% of F1 progeny in experimental settings [90]. VSNJV-infected *C. sonorensis* midges transmitted the virus to 76% of susceptible males, a fraction of which were able to transmit the virus back to naïve females [67]. However, rates of transmission by *L. shannoni* (20%) to F1 progeny are not believed to be sufficient for viral maintenance without a vertebrate reservoir [5]. This conclusion is supported by the disappearance of VSNJV on Ossabaw Island, USA, after the primary vertebrate host reservoir, *Sus scrofa* (feral swine), was removed [49]. Venereal transmission in *C. sonorensis* is more efficient, but VSNJV RNA was only detected in one out of 47 midge pools screened in our study during the dry season, and midge abundance declined significantly in the dry season. Susceptible black flies can also acquire the virus from infected black flies by co-feeding on the same host, although this has not been studied in the other insect taxa of interest [89].

Fourth, transmission from a wildlife reservoir may seed VSNJV-positive insects into ranches. In Mexico and other countries, neutralizing antibodies to VSV have been detected in a wide range of mammalian wildlife vertebrate host species in the orders Artiodactyla, Carnivora, Cingulata, Chiroptera, Didelphimorphia, Lagomorpha, Pilosa, Primates, and Rodentia [2,91,92,93,94,95]. However, detection of VSV-neutralizing antibodies only indicates prior exposure of the host but does not constitute evidence that the host serves as a reservoir of the virus [96]. Although VSV has not been isolated from a wildlife host from the field, laboratory experiments have reported that deer mice (*Peromyscus maniculatus*) can produce viremia and infect black flies [97,98]. These laboratory results and serosurveillance data suggest that a VSV wildlife reservoir may exist, but the gold standard of proof (virus isolation) remains to be met.

The proportion of VSNJV RNA-positive insects also varied significantly among LULC types, but this difference was seasonal. The highest rate of VSNJV carriage was observed in the stables during the rainy season. In the post-rainy season, the highest rate of VSNJV carriage was observed in both stables and pastures. Interestingly, no significant differences were detected during the dry season. Given that livestock host cases were reported during the rainy and post-rainy seasons, it is not surprising to find a significantly higher proportion of VSNJV-positive insect vectors in the area where cattle are most concentrated (stables and pastures). It has been previously reported that livestock are commonly infected by VSV in pastures, and this has been corroborated by oral accounts from the ranchers at our sites [2]. The lack of significant differences in VSV-positive insects among LULC types during the dry season implies a shift in the focus of VSV transmission.

The taxa predominantly carrying VSNJV RNA also varied by season. Sandflies showed the highest proportion of VSNJV positivity in the rainy season, but mosquitoes showed the highest proportion of VSNJV positivity in the dry season. These differences suggest a “baton pass” model in which different vectors may sustain VSV transmission in different seasons, acknowledging that VSV RNA carriage does not imply vector status. This hypothesis is supported by differences in relative abundance, with sand flies peaking in the 2021 rainy season and mosquitoes being relatively more abundant than other taxa in the dry season. McMillan et al. has also proposed a similar mechanism in Jamestown Canyon virus (JCV), where JCV overwinters in univoltine *Aedes* spp. mosquitoes, but multivoltine *Aedes* spp. act as amplifying vectors [99]. It is also possible that mosquito infections result from a cryptic wildlife cycle involving wildlife reservoirs with viremic stages in their infection that serve as a source for mosquitoes, in contrast with the largely non-viremic transmission cycle that occurs between livestock and other vectors (i.e., sandflies and blackflies).

The barcoding and phylogenetic analysis of VSNJV-positive insect samples identified a suite of twelve species (from three families) that were positive for VSNJV RNA. Previous insect sampling efforts in the enzootic region reported collections that mainly consisted of Culicidae (*Aedes* spp., *Anopheles* spp., *Coquillettidia* spp., *Culex* spp., *Haemagogus* spp., *Mansonia* spp., *Psorophora* spp.), and Ceratopogonidae (*Culicoides* spp.), while Simuliidae and Psychodidae (*Phlebotominae* spp.) were also collected [100]. However, these specimens were not screened for VSV [100]. Thus, our findings greatly expand the list of species known to be VSNJV RNA-positive and should motivate further VSV vector competence studies. In particular, *Culex* spp., *Culicoides insignis*, *Culicoides neopulicaris*, and *Lutzomyia cruciata* should be prioritized for such studies. *Culex* spp. were frequently VSNJV RNA-positive in the dry season, and *C. insignis* are confirmed vectors of bluetongue virus [101]. *L. cruciata* and *L. evansi* demonstrate zoophilic behavior, making them candidate VSV vectors [102], albeit the limited dispersal ability of sandflies may prevent them from transmitting the virus long distances [86]. Furthermore, our detection of VSV RNA in a variety of mosquito genera (*Aedes*, *Culex*, *Mansonia*, and *Psorophora*) underscores the need for further consideration of the potential role of this group in the VSV life cycle.

We also found support for the hypothesis that different insect species are involved in enzootic versus epizootic VSV transmission. In the United States, multiple species of black flies as well as *Culicoides* spp. midges were implicated in the 2020 VSIV epizootic [43,44]. These taxa, as well as *Lutzomyia* spp. sand flies, have also been implicated in prior VSNJV and VSIV epizootics in the United States [28,29,40,41,48]. In contrast, we detected VSNJV RNA in one pool of black flies from Chiapas, but black fly abundance was drastically lower than the other three taxa of interest. This suggests that black flies are unlikely to be the main arthropod vector responsible for endemic VSV transmission in this region. Although *Culicoides* spp. and *Lutzomyia* spp. have also been implicated in VSV outbreaks in the USA, we did not detect an overlap between the species identified in the USA and Chiapas. In the USA, the vectorial capacity of *Culicoides sonorensis* has been well documented, but other poorly studied *Culicoides* species have also tested positive for VSV (*C. stellifer*, *C. variipennis*, *C. selfia*) [5,26,40,67,103]. In contrast, our barcoded VSNJV-positive Ceratopogonidae samples consisted of *C. insignis*, *C. neopulicaris*, and *C. bambusicola.* We also detected VSNJV RNA in *L. evansi* and *L. cruciata*, but not in *L. shannoni* (one pool confirmed via barcode), which is a reported vector in both Panama and the Southeastern United States (Ossabaw Island) [30,48,49,104].

## 5. Conclusions

This study advances our understanding of endemic VSV transmission dynamics and motivates new hypotheses for the maintenance of endemic VSV during the dry season. Livestock case reports and livestock serosurveillance in five cattle ranches in Chiapas, Mexico, support previous reports that the force of infection of VSNJV is extremely high in the region, that livestock cases are confined to the rainy and post-rainy seasons, and that VSNJV is the predominant virus in the area. The relative abundance of the four insect taxa implicated as potential VSV vectors varied across seasons and LULC types, with sand flies predominating in stables in the rainy season and mosquitoes predominating in pastures and water sources in both the post-rainy and dry seasons. VSNJV was detected in all four taxa in all five ranches in all three sampling seasons, but different taxa showed a significantly higher proportion of infection in different seasons. This suggests that the environment in Chiapas permits year-round transmission of VSV and that such persistence may require a “baton-pass” of the virus among different vector taxa in different seasons. Detection of VSNJV RNA in the dry season, when no livestock cases were reported, suggests that virus maintenance during this season does not require livestock hosts and may involve the long-term survival of infected insects, transmission directly among insects, and/or contributions from an unidentified wildlife reservoir.

## Figures and Tables

**Table 1:** Ordered differences report for the effect of taxa and sampling season on sampled insect abundance (post hoc Tukey’s Honestly Significant Difference (HSD) test). Different letters denote statistically significant differences between levels (*p* < 0.05). Levels that share the same letter are not significantly different from each other.

Level	A	B	C	D	E	F	G	Least Square Mean (Number of Insects)
Ceratopogonidae, Rainy 2021	A							1.93
Culicidae, Post-Rainy 2021	A	B						1.92
Culicidae, Rainy 2021	A	B	C					1.83
Culicidae, Rainy 2022	A	B	C					1.81
Culicidae, Post-Rainy 2022	A	B	C	D				1.64
Psychodidae, Rainy 2021	A	B	C	D	E			1.44
Culicidae, Dry 2022	A	B	C	D	E			1.32
Ceratopogonidae, Rainy 2022	A	B	C	D	E	F		1.27
Psychodidae, Post-Rainy 2021		B	C	D	E	F		1.19
Ceratopogonidae, Post-Rainy 2021			C	D	E	F		1.18
Psychodidae, Dry 2022				D	E	F	G	0.97
Ceratopogonidae, Dry 2022					E	F	G	0.84
Ceratopogonidae, Post-Rainy 2022					E	F	G	0.72
Psychodidae, Post-Rainy 2022						F	G	0.54
Psychodidae, Rainy 2022							G	0.36

## Data Availability

The original data presented in the study are openly available at GitHub (https://github.com/zhoulhca/Zhou-Valdez_Viruses_Chiapas-VSV-Data (accessed on 3 August 2024). COI sequences for insect vectors are available at NCBI GenBank (Accession Numbers: PP815786-PP815876).

## Supplementary Materials

The following supporting information can be downloaded at: https://www.mdpi.com/article/10.3390/v16111742/s1 or https://github.com/zhoulhca/VSV_Vectors_Chiapas, Figure S1: Previous VSV vector studies in the endemic region. Circles indicate insect sampling and viral isolation from listed taxa; dotted squares represent insect sampling of listed taxa. Figure S2: Sampling sites by ranch and LULC type. Figure S3: Map of Ocozocoautla de Espinosa and sampled ranches. Images of all five ranches with trap locations are shown. Representative photos are shown for each LULC type. Figure S4: Representative document for the seasonal livestock case reporting survey distributed to ranchers. Figure S5: Insect sampling results by season (with *Psorophora* spp.). Daily average (±SEM) number of designated insect taxa sampled per ranch per sampling season. Results are colored by insect taxa and grouped by LULC type and season. Figure S6: Complete ML phylogeny for Ceratopogonidae; RAxML v.8, bootstrap values >75% (1000 replicates) are noted at the nodes. For generated barcodes, screening results are indicated with a red (VSNJV-positive) or blue (VSNJV-negative) circle, followed by the sample ID and morphological identification. GenBank sequences are displayed with accession numbers. Figure S7: Complete ML phylogeny for Culicidae (*Aedes* spp.); RAxML v.8, bootstrap values >75% (1000 replicates) are noted at the nodes. For generated barcodes, screening results are indicated with a red (VSNJV-positive) or blue (VSNJV-negative) circle, followed by the sample ID and morphological identification. GenBank sequences are displayed with accession numbers. Figure S8: Complete ML phylogeny for Culicidae (*Culex* spp.); RAxML v.8, bootstrap values >75% (1000 replicates) are noted at the nodes. For generated barcodes, screening results are indicated with a red (VSNJV-positive) or blue (VSNJV-negative) circle, followed by the sample ID and morphological identification. GenBank sequences are displayed with accession numbers. Table S1: Insect sampling sites (WGS84 GPS coordinates). Table S2: Primer and probe information used for VSV multiplex qRT-PCR screening and insect COI DNA barcoding primers. Table S3: GenBank sequences used for phylogenetic reconstruction (Species ID, Accession Number, Country of Origin). Table S4: Reported livestock VS cases metadata. Table S5: Livestock VSNJV neutralization assay titer information. Table S6: Barcode sequence pairwise identity tables for DNA barcodes. Supplementary File S1: Insect collection data by sampling season, taxa, and LULC. Supplementary File S2: Insect VSV screening data (qRT-PCR).
