# Supplementary material for: Vesicular Stomatitis Virus Transmission Dynamics Within Its Endemic Range in Chiapas, Mexico
Source: Viruses. 2024 Nov 6;16(11):1742. doi: 10.3390/v16111742 (PMC11598859; doi:10.3390/v16111742)
Supplement: Supplementary file 1 [file viruses-16-01742-s001.zip › Table S1.pdf]

Ranch	Site	Landcover/Land use Type	GPS Coordinates
Santa Clara del Roble	SCR_AP_01	Water Source	16.580250, -93.428000
Santa Clara del Roble	SCR_AP_02	Water Source	16.579833, -93.428056
Santa Clara del Roble	SCR_AP_03	Water Source	16.579639, -93.428111
Santa Clara del Roble	SCR_ET_01	Stable	16.580194, -93.429750
Santa Clara del Roble	SCR_ET_02	Stable	16.579889, -93.429639
Santa Clara del Roble	SCR_ET_03	Stable	16.580000, -93.429139
Santa Clara del Roble	SCR_PT_01	Pasture	16.583333, -93.434806
Santa Clara del Roble	SCR_PT_02	Pasture	16.583917, -93.436167
Santa Clara del Roble	SCR_PT_03	Pasture	16.584972, -93.437167
Kikapu	KIK_AP_01	Water Source	16.812778, -93.425500
Kikapu	KIK_AP_02	Water Source	16.812694, -93.425361
Kikapu	KIK_AP_03	Water Source	16.812806, -93.425278
Kikapu	KIK_ET_01	Stable	16.812750, -93.427028
Kikapu	KIK_ET_02	Stable	16.812139, -93.426250
Kikapu	KIK_ET_03	Stable	16.812472, -93.426750
Kikapu	KIK_PT_01	Pasture	16.808222, -93.432361
Kikapu	KIK_PT_02	Pasture	16.809028, -93.432694
Kikapu	KIK_PT_03	Pasture	16.807444, -93.433944
El Yaqui	REY_AP_01	Water Source	16.556278, -93.459306
El Yaqui	REY_AP_02	Water Source	16.556361, -93.459500
El Yaqui	REY_AP_03	Water Source	16.556111, -93.459667
El Yaqui	REY_ET_01	Stable	16.549861, -93.460472
El Yaqui	REY_ET_02	Stable	16.549583, -93.461083
El Yaqui	REY_ET_03	Stable	16.549111, -93.460333
El Yaqui	REY_PT_01	Pasture	16.556889, -93.459306
El Yaqui	REY_PT_02	Pasture	16.557833, -93.457750
El Yaqui	REY_PT_03	Pasture	16.557833, -93.460083
Raudal del Potro	RDP_AP_01	Water Source	16.621833, -93.442056
Raudal del Potro	RDP_AP_02	Water Source	16.621611, -93.443806
Raudal del Potro	RDP_AP_03	Water Source	16.621528, -93.444556
Raudal del Potro	RDP_ET_01	Stable	16.619778, -93.431611
Raudal del Potro	RDP_ET_02	Stable	16.619944, -93.431861
Raudal del Potro	RDP_ET_03	Stable	16.619694, -93.432111
Raudal del Potro	RDP_PT_01	Pasture	16.621361, -93.439861
Raudal del Potro	RDP_PT_02	Pasture	16.621917, -93.438722
Raudal del Potro	RDP_PT_03	Pasture	16.621028, -93.437028
Veinte Casas	20C_AP_01	Water Source	16.922250, -93.461944
Veinte Casas	20C_AP_02	Water Source	16.922306, -93.463250
Veinte Casas	20C_AP_03	Water Source	16.925333, -93.463028
Veinte Casas	20C_ET_01	Stable	16.926222, -93.464250
Veinte Casas	20C_ET_02	Stable	16.926444, -93.463861
Veinte Casas	20C_ET_03	Stable	16.926000, -93.464056

Veinte Casas	20C_PT_01	Pasture	16.923972, -93.461889
Veinte Casas	20C_PT_02	Pasture	16.923861, -93.461444
Veinte Casas	20C_PT_03	Pasture	16.923028, -93.461667
