# Supplementary material for: Vesicular Stomatitis Virus Transmission Dynamics Within Its Endemic Range in Chiapas, Mexico
Source: Viruses. 2024 Nov 6;16(11):1742. doi: 10.3390/v16111742 (PMC11598859; doi:10.3390/v16111742)
Supplement: Supplementary file 1 [file viruses-16-01742-s001.zip › Table S2.pdf]

Purpose	Type	Target	Amplicon Size	Name	Sequence
Virus Detection	Primer	VSV-IN L gene	227 bp	7230F	TGATACAGTACAATTATTTTGGGAC
Virus Detection	Primer	VSV-IN L gene	227 bp	7456R	GAGACTTTCTGTTACGGGATCTGG
Virus Detection	Primer	VSV-NJ L gene	266 bp	7230F-1	TGATTCAATATAATTATTTTGGGAC
Virus Detection	Primer	VSV-NJ L gene	266 bp	7230F-2	TGATTCAATATAATTACTTTGGAAC
Virus Detection	Primer	VSV-NJ L gene	266 bp	REV2	AGGCTCAGAGGCATGTTCAT
Virus Detection	Probe	VSV-IN	NA	VSV IN 22 probe	ATGATGCATGATCCTGCTCTTC
Virus Detection	Probe	VSV-NJ	NA	VSV NJ M1 probe	TTTATGCATGATCCCGCAATACG
Virus Detection	Probe	VSV-NJ	NA	VSV NJ M2 probe	TTTATGCATGACCCTGCCATAAG
Virus Detection	Probe	VSV-NJ	NA	VSV NJ probe (short)	TTGCACACCAGAACAT
Barcoding	Primer	COI gene	649 bp	LCO1490	GGTCAACAAATCATAAAGATATTGG
Barcoding	Primer	COI gene	649 bp	HCO2198	TAAACTTCAGGGTGACCAAAAAATCA
