# Supplementary material for: Vesicular Stomatitis Virus Transmission Dynamics Within Its Endemic Range in Chiapas, Mexico
Source: Viruses. 2024 Nov 6;16(11):1742. doi: 10.3390/v16111742 (PMC11598859; doi:10.3390/v16111742)
Supplement: Supplementary file 1 [file viruses-16-01742-s001.zip › Table S3.pdf]

Supplementary Table 3: Taxonomic information and Genbank Accession Numbers for COI sequences used for phylogenetic reconstruction and species identification

Family	Genus	*Species ID*	Genbank Accession Number	Country
Ceratopogonidae	Culicoides	*Culicoides (Avaritia) boydi*	OM892618.1	USA
Ceratopogonidae	Culicoides	*Culicoides (Avaritia) pusilloides*	OL619993.1	Brazil
Ceratopogonidae	Culicoides	*Culicoides (Avaritia) pusillus*	MK760235.1	France
Ceratopogonidae	Culicoides	*Culicoides (Beltranmyia) crepuscularis*	MT328934.1	USA
Ceratopogonidae	Culicoides	*Culicoides (Culicoides) neopulicaris*	KT794171.1	USA
Ceratopogonidae	Culicoides	*Culicoides (Diphaomyia) haematopotus*	MT328942.1	USA
Ceratopogonidae	Culicoides	*Culicoides (Diphaomyia) iriartei*	ON002337.1	Brazil
Ceratopogonidae	Culicoides	*Culicoides (Drymodesmyia) bakeri*	OM892587.1	USA
Ceratopogonidae	Culicoides	*Culicoides (Drymodesmyia) cacticola*	OM897428.1	USA
Ceratopogonidae	Culicoides	*Culicoides (Drymodesmyia) copiosus*	OM892637.1	USA
Ceratopogonidae	Culicoides	*Culicoides (Drymodesmyia) jamaicensis*	LC744012.1	Dominican Republic
Ceratopogonidae	Culicoides	*Culicoides (Drymodesmyia) ryckmani*	OM892646.1	USA
Ceratopogonidae	Culicoides	*Culicoides (Drymodesmyia) sitiens*	OM892636.1	USA
Ceratopogonidae	Culicoides	*Culicoides (Haematomyidium) debilipalpis*	OR030072.1	Brazil
Ceratopogonidae	Culicoides	*Culicoides (Haematomyidium) kettlei*	OM892599.1	USA
Ceratopogonidae	Culicoides	*Culicoides (Haematomyidium) paraensis*	OR030070.1	Brazil
Ceratopogonidae	Culicoides	*Culicoides (Hoffmania) diabolicus*	KY707783	France
Ceratopogonidae	Culicoides	*Culicoides (Hoffmania) foxi*	OR030056.1	Brazil
Ceratopogonidae	Culicoides	*Culicoides (Hoffmania) hylas*	OP088717.1	Brazil
Ceratopogonidae	Culicoides	*Culicoides (Hoffmania) insignis*	MW871560.1	Brazil
Ceratopogonidae	Culicoides	*Culicoides (Monoculicoides) occidentalis*	KY707882.1	USA
Ceratopogonidae	Culicoides	*Culicoides (Monoculicoides) sonorensis*	OL604717.1	USA
Ceratopogonidae	Culicoides	*Culicoides (Monoculicoides) variipennis*	KT794141.1	USA
Ceratopogonidae	Culicoides	*Culicoides (Oecacta) furens*	ON002322.1	Brazil
Ceratopogonidae	Culicoides	*Culicoides (Selfia) multipunctatus*	KT794154.1	USA
Ceratopogonidae	Culicoides	*Culicoides propriipennis*	OM704795.1	Brazil

Ceratopogonidae	Culicoides	*Culicoides leopoldoi*	OR030074.1	Brazil
Ceratopogonidae	Culicoides	*Culicoides glabellus*	OM704764.1	Brazil
Ceratopogonidae	Culicoides	*Culicoides luglani*	OM892613.1	USA
Ceratopogonidae	Culicoides	*Culicoides mohave*	OM892605.1	USA
Ceratopogonidae	Culicoides	*Culicoides bambusicola*	OL619984.1	Brazil
Psychodidae	Brumptomyia	*Brumptomyia hamata*	MT333244.1	Colombia
Psychodidae	Brumptomyia	*Brumptomyia mesai*	MK851244.1	Mexico
Psychodidae	Dampfoymia	*Dampfomyia (Coromyia) beltrani*	OP781332.1	Mexico
Psychodidae	Dampfoymia	*Dampfomyia (Coromyia) deleoni*	MK851253.1	Mexico
Psychodidae	Dampfoymia	*Dampfomyia (Dampfomyia) anthophora*	MK952678.1	USA
Psychodidae	Lutzomyia	*Lutzomyia (Helcocyrtomyia) hartmanni*	KC921258.1	Colombia
Psychodidae	Lutzomyia	*Lutzomyia (Lutzomyia) longipalpis*	MW535726.1	Honduras
Psychodidae	Lutzomyia	*Lutzomyia (Tricholateralis) cruciata*	OQ325337.1	Mexico
Psychodidae	Lutzomyia	*Lutzomyia (Tricholateralis) gomezi*	KC921254.1	Colombia
Psychodidae	Lutzomyia	*Lutzomyia (Pifanomyia) evansi*	OP964305.1	Brazil
Psychodidae	Pintomyia	*Pintomyia (Pifanomyia) ovallesi*	MN257603.1	Panama
Psychodidae	Pintomyia	*Pintomyia (Pifanomyia) serrana*	OR555674.1	Brazil
Psychodidae	Trichopygomyia	*Trichopygomyia triramula*	KC921316.1	Colombia
Psychodidae	Bichromomyia	*Bichromomyia olmeca olmeca*	MN257587.1	Panama
Psychodidae	Nyssomyia	*Nyssomyia ylephiletor*	OP784411.1	Mexico
Psychodidae	Psathyromyia	*Psathyromyia (Forattiniella) carpenteri*	OQ325338.1	Mexico
Psychodidae	Psathyromyia	*Psathyromyia (Forattiniella) texana*	MK952614.1	USA
Psychodidae	Psathyromyia	*Psathyromyia (Psathyromyia) cratifer*	OR608122.1	Mexico
Psychodidae	Psathyromyia	*Lutzomyia (Psathyromyia) shannoni*	KC755396.1	Mexico
Psychodidae	Psathyromyia	*Psathyromyia maya*	OP784398.1	Mexico
Psychodidae	Psathyromyia	*Psathyromyia (Xyphopsathyromyia) aclydifera*	GU001725.1	Panama
Psychodidae	Psychodopygus	*Psychodopygus panamensis*	OP784416.1	Mexico
Psychodidae	Micropygomyia	*Micropygomyia cayennensis*	MT338251.1	Colombia
Psychodidae	Micropygomyia	*Micropygomyia cayennensis cayennensis*	GU909476.1	Colombia
Psychodidae	Micropygomyia	*Micropygomyia (Sauromyia) trinidadensis*	OP964258.1	Colombia

Psychodidae	Clogmia	*Clogmia albipunctata*	GU803880.1	USA
Psychodidae	Psychoda	*Psychoda (Psychodocha) cinerea*	MF855905.1	Canada
Psychodidae	Psychoda	*Psychoda (Tinearia) alternata*	HM433828.1	Canada
Culicidae	Aedes	*Aedes aegypti*	OQ352574.1	Mexico
Culicidae	Aedes	*Aedes albopictus*	OP786865.1	USA
Culicidae	Aedes	*Aedes allotecnon*	MT552571.1	Mexico
Culicidae	Aedes	*Aedes angustivittatus*	MT999212.1	Mexico
Culicidae	Aedes	*Aedes brelandi*	MG242473.1	USA
Culicidae	Aedes	*Aedes burgeri*	JX259536.1	USA
Culicidae	Aedes	*Aedes campestris*	KR746992.1	Canada
Culicidae	Aedes	*Aedes canadensis*	JX259543.1	USA
Culicidae	Aedes	*Aedes chionotum*	MT108606.1	Mexico
Culicidae	Aedes	*Aedes cozumelensis*	MW339680.1	Mexico
Culicidae	Aedes	*Aedes dorsalis*	JX259592.1	USA
Culicidae	Aedes	*Aedes dupreei*	MG242489.1	USA
Culicidae	Aedes	*Aedes epactius*	MT999294.1	Mexico
Culicidae	Aedes	*Aedes euplocamus*	MN968255.1	MX
Culicidae	Aedes	*Aedes fluviatilis*	MT999307.1	Mexico
Culicidae	Aedes	*Aedes fulvus*	MT552438.1	Mexico
Culicidae	Aedes	*Aedes gabriel*	MT108655.1	Mexico
Culicidae	Aedes	*Aedes guatemala*	MT999329.1	Mexico
Culicidae	Aedes	*Aedes guerrero*	MT108603.1	Mexico
Culicidae	Aedes	*Aedes hastatus*	MW363436.1	Argentina
Culicidae	Aedes	*Aedes infirmatus*	JX259638.1	USA
Culicidae	Aedes	*Aedes insolitus*	MT552439.1	Mexico
Culicidae	Aedes	*Aedes lorraineae*	MT999309.1	Mexico
Culicidae	Aedes	*Aedes melanimon*	JX259647.1	USA
Culicidae	Aedes	*Aedes mitchellae*	MG242507.1	USA
Culicidae	Aedes	*Aedes monticola*	JX259649.1	USA
Culicidae	Aedes	*Aedes nigromaculis*	JX260446.1	USA
Culicidae	Aedes	*Aedes podographicus*	MT999338.1	Mexico

Culicidae	Aedes	*Aedes purpureipes*	JX260451.1	USA
Culicidae	Aedes	*Aedes quadrivittatus*	MT999305.1	Mexico
Culicidae	Aedes	*Aedes ramirezi*	MT108590.1	Mexico
Culicidae	Aedes	*Aedes sandrae*	MT999244.1	Mexico
Culicidae	Aedes	*Aedes serratus*	MW339694.1	Mexico
Culicidae	Aedes	*Aedes spencerii*	JX259672.1	USA
Culicidae	Aedes	*Aedes squamiger*	JX259675.1	USA
Culicidae	Aedes	*Aedes taeniorhynchus*	MW339699.1	Mexico
Culicidae	Aedes	*Aedes tehuantepec*	MT999292.1	Mexico
Culicidae	Aedes	*Aedes thelcter*	JX259680.1	USA
Culicidae	Aedes	*Aedes tortilis*	JX259683.1	USA
Culicidae	Aedes	*Aedes triseriatus*	JX260473.1	USA
Culicidae	Aedes	*Aedes trivittatus*	MT108662.1	Mexico
Culicidae	Aedes	*Aedes vargasi*	MT108667.1	Mexico
Culicidae	Aedes	*Aedes vexans*	OP730900.1	USA
Culicidae	Aedes	*Aedes zoosophus*	JX259710.1	USA
Culicidae	Anopheles	*Anopheles albimanus*	OL473478.1	Honduras
Culicidae	Anopheles	*Anopheles apicimacula*	OL473784.1	Honduras
Culicidae	Anopheles	*Anopheles argyritarsis*	OL471425.1	Honduras
Culicidae	Anopheles	*Anopheles crucians*	OP729921.1	USA
Culicidae	Anopheles	*Anopheles darlingi*	MT034037.1	Honduras
Culicidae	Anopheles	*Anopheles eiseni*	MT999254.1	Mexico
Culicidae	Anopheles	*Anopheles neivai*	PP277450.1	Colombia
Culicidae	Anopheles	*Anopheles neomaculipalpus*	OL473769.1	Honduras
Culicidae	Anopheles	*Anopheles pseudopunctipennis*	MT999248.1	Mexico
Culicidae	Anopheles	*Anopheles punctipennis*	OP727306.1	USA
Culicidae	Anopheles	*Anopheles quadrimaculatus*	OP830855.1	USA
Culicidae	Anopheles	*Anopheles veruslanei*	MW339707.1	Mexico
Culicidae	Anopheles	*Anopheles vestitipennis*	MN968269.1	Mexico
Culicidae	Anopheles	*Anopheles walkeri*	JF868964.1	Canada
Culicidae	Coquillettidia	*Coquillettidia nigrcans*	MN968281.1	Mexico

Culicidae	Coquillettidia	*Coquillettidia perturbans*	OP853412.1	USA
Culicidae	Coquillettidia	*Coquillettidia venezuelensis*	OP785620.1	Brazil
Culicidae	Culex	*Culex apicalis*	JX259906.1	USA
Culicidae	Culex	*Culex arizonensis*	MT108554.1	Mexico
Culicidae	Culex	*Culex bastagarius*	MW363414.1	Argentina
Culicidae	Culex	*Culex bidens*	MH931448.1	Colombia
Culicidae	Culex	*Culex bihaicola*	MT999256.1	Mexico
Culicidae	Culex	*Culex chidesteri*	KF919253.	Brazil
Culicidae	Culex	*Culex conservator*	KF671017.1	Ecuador
Culicidae	Culex	*Culex conspirator*	KM593054.1	Colombia
Culicidae	Culex	*Culex corniger*	MT999314.1	Mexico
Culicidae	Culex	*Culex coronator*	MT999227.1	Mexico
Culicidae	Culex	*Culex crybda*	MT999312.1	Mexico
Culicidae	Culex	*Culex declarator*	MT999325.1	Mexico
Culicidae	Culex	*Culex dunni*	KX779796.1	Brazil
Culicidae	Culex	*Culex eastor*	MT552527.1	Mexico
Culicidae	Culex	*Culex educator*	KM593042.1	Colombia
Culicidae	Culex	*Culex elevator*	MT999336.1	Mexico
Culicidae	Culex	*Culex erraticus*	MN968247.1	Mexico
Culicidae	Culex	*Culex erythrothorax*	KM593011.1	Colombia
Culicidae	Culex	*Culex imitator*	GU291982.1	Brazil
Culicidae	Culex	*Culex inhibitator*	JX259908.1	USA
Culicidae	Culex	*Culex interrogator*	MT999234.1	Mexico
Culicidae	Culex	*Culex lactator*	KM593012.1	Colombia
Culicidae	Culex	*Culex mollis*	MT999337.1	Mexico
Culicidae	Culex	*Culex nigripalpus*	MW339716.1	Mexico
Culicidae	Culex	*Culex pedroi*	KX379627.1	Brazil
Culicidae	Culex	*Culex pilosus*	MT552534.1	Mexico
Culicidae	Culex	*Culex pinarocampa*	MW057167.1	Mexico
Culicidae	Culex	*Culex pipens*	PP140837.1	Bangladesh
Culicidae	Culex	*Culex pseudostigmatosoma*	MT108678.1	Mexico

Culicidae	Culex	*Culex quinquefasciatus*	MW509603.1	Puerto Rico
Culicidae	Culex	*Culex rejector*	MT108586.1	Mexico
Culicidae	Culex	*Culex restrictor*	MT552397.1	Mexico
Culicidae	Culex	*Culex restuans*	MT108676.1	Mexico
Culicidae	Culex	*Culex salinarius*	MT108593.1	Mexico
Culicidae	Culex	*Culex spissipes*	KX779870.1	Brazil
Culicidae	Culex	*Culex stigmatosoma*	MT108615.1	Mexico
Culicidae	Culex	*Culex taeniopus*	MW339726.1	Mexico
Culicidae	Culex	*Culex tarsalis*	MT108659.1	Mexico
Culicidae	Culex	*Culex theobaldi*	MN997415.1	Colombia
Culicidae	Culex	*Culex thriambus*	MT108663.1	Mexico
Culicidae	Culex	*Culex usquatus*	MT552583.1	Mexico
Culicidae	Haemagogus	*Haemagogus equinus*	MW339727.1	Mexico
Culicidae	Haemagogus	*Haemagogus mesodentatus*	MT999321.1	Mexico
Culicidae	Sabethes	*Sabethes chloropterus*	MT999310.1	Mexico
Culicidae	Sabethes	*Sabethes cyaneus*	MT418579.1	Colombia
Culicidae	Wyeomyia	*Wyeomyia abebela*	MT999298.1	Mexico
Culicidae	Wyeomyia	*Wyeomyia adelpha*	MT999332.1	Mexico
Culicidae	Wyeomyia	*Wyeomyia aporonoma*	MT999306.1	Mexico
Culicidae	Wyeomyia	*Wyeomyia arthrostigma*	PP372857.1	Colombia
Culicidae	Wyeomyia	*Wyeomyia celaenocephala*	MW339754.1	Mexico
Culicidae	Wyeomyia	*Wyeomyia guatemala/adelpha*	MT552365.1	Mexico
Culicidae	Wyeomyia	*Wyeomyia melanopus*	MT552426.1	Mexico
Culicidae	Wyeomyia	*Wyeomyia pertinans*	MN997648.1	Colombia
Culicidae	Wyeomyia	*Wyeomyia pseudopecten*	MT552368.1	Mexico

Family	Genus	*Species ID*	Genbank Accession Number	Country
Ceratopogonidae	Culicoides	*Culicoides (Avaritia) boydi*	OM892618.1	USA
Ceratopogonidae	Culicoides	*Culicoides (Avaritia) pusilloides*	OL619993.1	Brazil
Ceratopogonidae	Culicoides	*Culicoides (Avaritia) pusillus*	MK760235.1	France
Ceratopogonidae	Culicoides	*Culicoides (Beltranmyia) crepuscularis*	MT328934.1	USA
Ceratopogonidae	Culicoides	*Culicoides (Culicoides) neopulicaris*	KT794171.1	USA
Ceratopogonidae	Culicoides	*Culicoides (Diphaomyia) haematopotus*	MT328942.1	USA
Ceratopogonidae	Culicoides	*Culicoides (Diphaomyia) iriartei*	ON002337.1	Brazil
Ceratopogonidae	Culicoides	*Culicoides (Drymodesmyia) bakeri*	OM892587.1	USA
Ceratopogonidae	Culicoides	*Culicoides (Drymodesmyia) cacticola*	OM897428.1	USA
Ceratopogonidae	Culicoides	*Culicoides (Drymodesmyia) copiosus*	OM892637.1	USA
Ceratopogonidae	Culicoides	*Culicoides (Drymodesmyia) jamaicensis*	LC744012.1	Dominican Republic
Ceratopogonidae	Culicoides	*Culicoides (Drymodesmyia) ryckmani*	OM892646.1	USA
Ceratopogonidae	Culicoides	*Culicoides (Drymodesmyia) sitiens*	OM892636.1	USA
Ceratopogonidae	Culicoides	*Culicoides (Haematomyidium) debilipalpis*	OR030072.1	Brazil
Ceratopogonidae	Culicoides	*Culicoides (Haematomyidium) kettlei*	OM892599.1	USA
Ceratopogonidae	Culicoides	*Culicoides (Haematomyidium) paraensis*	OR030070.1	Brazil
Ceratopogonidae	Culicoides	*Culicoides (Hoffmania) diabolicus*	KY707783	France
Ceratopogonidae	Culicoides	*Culicoides (Hoffmania) foxi*	OR030056.1	Brazil
Ceratopogonidae	Culicoides	*Culicoides (Hoffmania) hylas*	OP088717.1	Brazil
Ceratopogonidae	Culicoides	*Culicoides (Hoffmania) insignis*	MW871560.1	Brazil
Ceratopogonidae	Culicoides	*Culicoides (Monoculicoides) occidentalis*	KY707882.1	USA
Ceratopogonidae	Culicoides	*Culicoides (Monoculicoides) sonorensis*	OL604717.1	USA
Ceratopogonidae	Culicoides	*Culicoides (Monoculicoides) variipennis*	KT794141.1	USA
Ceratopogonidae	Culicoides	*Culicoides (Oecacta) furens*	ON002322.1	Brazil
Ceratopogonidae	Culicoides	*Culicoides (Selfia) multipunctatus*	KT794154.1	USA
Ceratopogonidae	Culicoides	*Culicoides propriipennis*	OM704795.1	Brazil
Ceratopogonidae	Culicoides	*Culicoides leopoldoi*	OR030074.1	Brazil
Ceratopogonidae	Culicoides	*Culicoides glabellus*	OM704764.1	Brazil

Ceratopogonidae	Culicoides	*Culicoides luglani*	OM892613.1	USA
Ceratopogonidae	Culicoides	*Culicoides mohave*	OM892605.1	USA
Ceratopogonidae	Culicoides	*Culicoides bambusicola*	OL619984.1	Brazil
Psychodidae	Brumptomyia	*Brumptomyia hamata*	MT333244.1	Colombia
Psychodidae	Brumptomyia	*Brumptomyia mesai*	MK851244.1	Mexico
Psychodidae	Dampfoymia	*Dampfomyia (Coromyia) beltrani*	OP781332.1	Mexico
Psychodidae	Dampfoymia	*Dampfomyia (Coromyia) deleoni*	MK851253.1	Mexico
Psychodidae	Dampfoymia	*Dampfomyia (Dampfomyia) anthophora*	MK952678.1	USA
Psychodidae	Lutzomyia	*Lutzomyia (Helcocyrtomyia) hartmanni*	KC921258.1	Colombia
Psychodidae	Lutzomyia	*Lutzomyia (Lutzomyia) longipalpis*	MW535726.1	Honduras
Psychodidae	Lutzomyia	*Lutzomyia (Tricholateralis) cruciata*	OQ325337.1	Mexico
Psychodidae	Lutzomyia	*Lutzomyia (Tricholateralis) gomezi*	KC921254.1	Colombia
Psychodidae	Lutzomyia	*Lutzomyia (Pifanomyia) evansi*	OP964305.1	Brazil
Psychodidae	Pintomyia	*Pintomyia (Pifanomyia) ovallesi*	MN257603.1	Panama
Psychodidae	Pintomyia	*Pintomyia (Pifanomyia) serrana*	OR555674.1	Brazil
Psychodidae	Trichopygomyia	*Trichopygomyia triramula*	KC921316.1	Colombia
Psychodidae	Bichromomyia	*Bichromomyia olmeca olmeca*	MN257587.1	Panama
Psychodidae	Nyssomyia	*Nyssomyia ylephiletor*	OP784411.1	Mexico
Psychodidae	Psathyromyia	*Psathyromyia (Forattiniella) carpenteri*	OQ325338.1	Mexico
Psychodidae	Psathyromyia	*Psathyromyia (Forattiniella) texana*	MK952614.1	USA
Psychodidae	Psathyromyia	*Psathyromyia (Psathyromyia) cratifer*	OR608122.1	Mexico
Psychodidae	Psathyromyia	*Lutzomyia (Psathyromyia) shannoni*	KC755396.1	Mexico
Psychodidae	Psathyromyia	*Psathyromyia maya*	OP784398.1	Mexico
Psychodidae	Psathyromyia	*Psathyromyia (Xyphopsathyromyia) aclydifera*	GU001725.1	Panama
Psychodidae	Psychodopygus	*Psychodopygus panamensis*	OP784416.1	Mexico
Psychodidae	Micropygomyia	*Micropygomyia cayennensis*	MT338251.1	Colombia
Psychodidae	Micropygomyia	*Micropygomyia cayennensis cayennensis*	GU909476.1	Colombia
Psychodidae	Micropygomyia	*Micropygomyia (Sauromyia) trinidadensis*	OP964258.1	Colombia
Psychodidae	Clogmia	*Clogmia albipunctata*	GU803880.1	USA

Psychodidae	Psychoda	*Psychoda (Psychodocha) cinerea*	MF855905.1	Canada
Psychodidae	Psychoda	*Psychoda (Tinearia) alternata*	HM433828.1	Canada
Culicidae	Aedes	*Aedes aegypti*	OQ352574.1	Mexico
Culicidae	Aedes	*Aedes albopictus*	OP786865.1	USA
Culicidae	Aedes	*Aedes allotecnon*	MT552571.1	Mexico
Culicidae	Aedes	*Aedes angustivittatus*	MT999212.1	Mexico
Culicidae	Aedes	*Aedes brelandi*	MG242473.1	USA
Culicidae	Aedes	*Aedes burgeri*	JX259536.1	USA
Culicidae	Aedes	*Aedes campestris*	KR746992.1	Canada
Culicidae	Aedes	*Aedes canadensis*	JX259543.1	USA
Culicidae	Aedes	*Aedes chionotum*	MT108606.1	Mexico
Culicidae	Aedes	*Aedes cozumelensis*	MW339680.1	Mexico
Culicidae	Aedes	*Aedes dorsalis*	JX259592.1	USA
Culicidae	Aedes	*Aedes dupreei*	MG242489.1	USA
Culicidae	Aedes	*Aedes epactius*	MT999294.1	Mexico
Culicidae	Aedes	*Aedes euplocamus*	MN968255.1	MX
Culicidae	Aedes	*Aedes fluviatilis*	MT999307.1	Mexico
Culicidae	Aedes	*Aedes fulvus*	MT552438.1	Mexico
Culicidae	Aedes	*Aedes gabriel*	MT108655.1	Mexico
Culicidae	Aedes	*Aedes guatemala*	MT999329.1	Mexico
Culicidae	Aedes	*Aedes guerrero*	MT108603.1	Mexico
Culicidae	Aedes	*Aedes hastatus*	MW363436.1	Argentina
Culicidae	Aedes	*Aedes infirmatus*	JX259638.1	USA
Culicidae	Aedes	*Aedes insolitus*	MT552439.1	Mexico
Culicidae	Aedes	*Aedes lorraineae*	MT999309.1	Mexico
Culicidae	Aedes	*Aedes melanimon*	JX259647.1	USA
Culicidae	Aedes	*Aedes mitchellae*	MG242507.1	USA
Culicidae	Aedes	*Aedes monticola*	JX259649.1	USA
Culicidae	Aedes	*Aedes nigromaculis*	JX260446.1	USA
Culicidae	Aedes	*Aedes podographicus*	MT999338.1	Mexico
Culicidae	Aedes	*Aedes purpureipes*	JX260451.1	USA

Culicidae	Aedes	*Aedes quadrivittatus*	MT999305.1	Mexico
Culicidae	Aedes	*Aedes ramirezi*	MT108590.1	Mexico
Culicidae	Aedes	*Aedes sandrae*	MT999244.1	Mexico
Culicidae	Aedes	*Aedes serratus*	MW339694.1	Mexico
Culicidae	Aedes	*Aedes spencerii*	JX259672.1	USA
Culicidae	Aedes	*Aedes squamiger*	JX259675.1	USA
Culicidae	Aedes	*Aedes taeniorhynchus*	MW339699.1	Mexico
Culicidae	Aedes	*Aedes tehuantepec*	MT999292.1	Mexico
Culicidae	Aedes	*Aedes thelcter*	JX259680.1	USA
Culicidae	Aedes	*Aedes tortilis*	JX259683.1	USA
Culicidae	Aedes	*Aedes triseriatus*	JX260473.1	USA
Culicidae	Aedes	*Aedes trivittatus*	MT108662.1	Mexico
Culicidae	Aedes	*Aedes vargasi*	MT108667.1	Mexico
Culicidae	Aedes	*Aedes vexans*	OP730900.1	USA
Culicidae	Aedes	*Aedes zoosophus*	JX259710.1	USA
Culicidae	Anopheles	*Anopheles albimanus*	OL473478.1	Honduras
Culicidae	Anopheles	*Anopheles apicimacula*	OL473784.1	Honduras
Culicidae	Anopheles	*Anopheles argyritarsis*	OL471425.1	Honduras
Culicidae	Anopheles	*Anopheles crucians*	OP729921.1	USA
Culicidae	Anopheles	*Anopheles darlingi*	MT034037.1	Honduras
Culicidae	Anopheles	*Anopheles eiseni*	MT999254.1	Mexico
Culicidae	Anopheles	*Anopheles neivai*	PP277450.1	Colombia
Culicidae	Anopheles	*Anopheles neomaculipalpus*	OL473769.1	Honduras
Culicidae	Anopheles	*Anopheles pseudopunctipennis*	MT999248.1	Mexico
Culicidae	Anopheles	*Anopheles punctipennis*	OP727306.1	USA
Culicidae	Anopheles	*Anopheles quadrimaculatus*	OP830855.1	USA
Culicidae	Anopheles	*Anopheles veruslanei*	MW339707.1	Mexico
Culicidae	Anopheles	*Anopheles vestitipennis*	MN968269.1	Mexico
Culicidae	Anopheles	*Anopheles walkeri*	JF868964.1	Canada
Culicidae	Coquillettidia	*Coquillettidia nigrcans*	MN968281.1	Mexico
Culicidae	Coquillettidia	*Coquillettidia perturbans*	OP853412.1	USA

Culicidae	Coquillettidia	*Coquillettidia venezuelensis*	OP785620.1	Brazil
Culicidae	Culex	*Culex apicalis*	JX259906.1	USA
Culicidae	Culex	*Culex arizonensis*	MT108554.1	Mexico
Culicidae	Culex	*Culex bastagarius*	MW363414.1	Argentina
Culicidae	Culex	*Culex bidens*	MH931448.1	Colombia
Culicidae	Culex	*Culex bihaicola*	MT999256.1	Mexico
Culicidae	Culex	*Culex chidesteri*	KF919253.	Brazil
Culicidae	Culex	*Culex conservator*	KF671017.1	Ecuador
Culicidae	Culex	*Culex conspirator*	KM593054.1	Colombia
Culicidae	Culex	*Culex corniger*	MT999314.1	Mexico
Culicidae	Culex	*Culex coronator*	MT999227.1	Mexico
Culicidae	Culex	*Culex crybda*	MT999312.1	Mexico
Culicidae	Culex	*Culex declarator*	MT999325.1	Mexico
Culicidae	Culex	*Culex dunni*	KX779796.1	Brazil
Culicidae	Culex	*Culex eastor*	MT552527.1	Mexico
Culicidae	Culex	*Culex educator*	KM593042.1	Colombia
Culicidae	Culex	*Culex elevator*	MT999336.1	Mexico
Culicidae	Culex	*Culex erraticus*	MN968247.1	Mexico
Culicidae	Culex	*Culex erythrothorax*	KM593011.1	Colombia
Culicidae	Culex	*Culex imitator*	GU291982.1	Brazil
Culicidae	Culex	*Culex inhibitator*	JX259908.1	USA
Culicidae	Culex	*Culex interrogator*	MT999234.1	Mexico
Culicidae	Culex	*Culex lactator*	KM593012.1	Colombia
Culicidae	Culex	*Culex mollis*	MT999337.1	Mexico
Culicidae	Culex	*Culex nigripalpus*	MW339716.1	Mexico
Culicidae	Culex	*Culex pedroi*	KX379627.1	Brazil
Culicidae	Culex	*Culex pilosus*	MT552534.1	Mexico
Culicidae	Culex	*Culex pinarocampa*	MW057167.1	Mexico
Culicidae	Culex	*Culex pipens*	PP140837.1	Bangladesh
Culicidae	Culex	*Culex pseudostigmatosoma*	MT108678.1	Mexico
Culicidae	Culex	*Culex quinquefasciatus*	MW509603.1	Puerto Rico

Culicidae	Culex	*Culex rejector*	MT108586.1	Mexico
Culicidae	Culex	*Culex restrictor*	MT552397.1	Mexico
Culicidae	Culex	*Culex restuans*	MT108676.1	Mexico
Culicidae	Culex	*Culex salinarius*	MT108593.1	Mexico
Culicidae	Culex	*Culex spissipes*	KX779870.1	Brazil
Culicidae	Culex	*Culex stigmatosoma*	MT108615.1	Mexico
Culicidae	Culex	*Culex taeniopus*	MW339726.1	Mexico
Culicidae	Culex	*Culex tarsalis*	MT108659.1	Mexico
Culicidae	Culex	*Culex theobaldi*	MN997415.1	Colombia
Culicidae	Culex	*Culex thriambus*	MT108663.1	Mexico
Culicidae	Culex	*Culex usquatus*	MT552583.1	Mexico
Culicidae	Haemagogus	*Haemagogus equinus*	MW339727.1	Mexico
Culicidae	Haemagogus	*Haemagogus mesodentatus*	MT999321.1	Mexico
Culicidae	Sabethes	*Sabethes chloropterus*	MT999310.1	Mexico
Culicidae	Sabethes	*Sabethes cyaneus*	MT418579.1	Colombia
Culicidae	Wyeomyia	*Wyeomyia abebela*	MT999298.1	Mexico
Culicidae	Wyeomyia	*Wyeomyia adelpha*	MT999332.1	Mexico
Culicidae	Wyeomyia	*Wyeomyia aporonoma*	MT999306.1	Mexico
Culicidae	Wyeomyia	*Wyeomyia arthrostigma*	PP372857.1	Colombia
Culicidae	Wyeomyia	*Wyeomyia celaenocephala*	MW339754.1	Mexico
Culicidae	Wyeomyia	*Wyeomyia guatemala/adelpha*	MT552365.1	Mexico
Culicidae	Wyeomyia	*Wyeomyia melanopus*	MT552426.1	Mexico
Culicidae	Wyeomyia	*Wyeomyia pertinans*	MN997648.1	Colombia
Culicidae	Wyeomyia	*Wyeomyia pseudopecten*	MT552368.1	Mexico
