# Supplementary material for: Vesicular Stomatitis Virus Transmission Dynamics Within Its Endemic Range in Chiapas, Mexico
Source: Viruses. 2024 Nov 6;16(11):1742. doi: 10.3390/v16111742 (PMC11598859; doi:10.3390/v16111742)
Supplement: Supplementary file 1 [file viruses-16-01742-s001.zip › Table S4.pdf]

Ranch	Date (mm/dd/yyyy)	Age	Animal	Sex	Reproductive State (Pregnant or not?)	Acquired from other property?	Symptoms and Notes
Santa Clara del Roble	5/14/2021	2 Years	Cow	Female	Not pregnant	No	Lesions in mouth and lower lip
Raudal del Potro	6/30/2021	15 Days	Cow	Female	Not pregnant	No	Lesions in mouth
Raudal del Potro	6/30/2021	12 Months	Cow	Female	Not pregnant	No	Lesions in mouth
Raudal del Potro	6/30/2021	3 Years	Cow	Female	Not pregnant	No	Lesions in mouth
Raudal del Potro*	6/30/2021	6 Years	Cow	Female	Not pregnant	No	Lesions in mouth
Raudal del Potro	6/30/2021	3 Years	Cow	Female	Not pregnant	No	Lesions in mouth and foot
Raudal del Potro	6/30/2021	6 Years	Cow	Female	Not pregnant	No	Lesions in mouth and foot
Raudal del Potro	6/30/2021	6 Months	Cow	Female	Not pregnant	No	Lesions in mouth
Kikapu	7/15/2021	Adult	Cow	Female	Pregnant (1st trimester)	No	Lesions on foot
Kikapu	8/6/2021	Adult	Cow	Female	Pregnant (1st trimester)	No	Lesions on foot
El Yaqui	8/10/2021	10 Years	Cow	Female	Not pregnant	Yes	Lesions on foot
El Yaqui	8/10/2021	6 Years	Cow	Female	Not pregnant	No	Lesions on foot
El Yaqui	8/10/2021	6 Years	Cow	Female	Not pregnant	No	Lesions on foot
Santa Clara del Roble	8/27/2021	5 Years	Cow	Female	Not pregnant (lactating)	No	Epithelial shedding in the nose
Kikapu	9/2/2021	Adult	Cow	Female	Pregnant (1st trimester)	No	Lesions on foot
Kikapu	9/4/2021	Adult	Cow	Female	Pregnant (2nd trimester)	No	Lesions on foot

El Yaqui	9/5/2021	6 Years	Cow	Male	Not pregnant	Yes	Lesions on foot
El Yaqui	9/5/2021	8 Years	Cow	Female	Not pregnant	No	Lesions on foot
El Yaqui	9/8/2021	11 Years	Cow	Female	Not pregnant	Yes	Lesions on foot
El Yaqui	9/15/2021	5 Years	Cow	Male	Not pregnant	No	Lesions on foot
Santa Clara del Roble	11/2/2021	5 Years	Cow	Female	N/A	No	Lesions in mouth
Raudal del Potro*	11/10/2021	6 Years	Cow	Female	Not pregnant	No	Lesions on foot<br>Lesions in mouth
Raudal del Potro	6/1/2022	8 Years	Cow	Female	Not pregnant	No	Lesions in mouth (tongue)
Raudal del Potro	6/1/2022	4 Years	Cow	Female	Not pregnant	No	Lesions in mouth (tongue)
Raudal del Potro	6/1/2022	4 Years	Cow	Female	Not pregnant	No	Lesions in mouth (tongue)
Santa Clara del Roble	7/25/2022	15 Months	Cow	Female	Not pregnant	No	Excessive salivation and lesions in mouth
Santa Clara del Roble	7/29/2022	11 Months	Cow	Female	Not pregnant	No	Excessive salivation and lesions in mouth
El Yaqui	11/2/2022	6 Years	Cow	Female	Not pregnant	No	Lesions in mouth

* = Re-infection case (highlighted)
