# Supplementary material for: Vesicular Stomatitis Virus Transmission Dynamics Within Its Endemic Range in Chiapas, Mexico
Source: Viruses. 2024 Nov 6;16(11):1742. doi: 10.3390/v16111742 (PMC11598859; doi:10.3390/v16111742)
Supplement: Supplementary file 1 [file viruses-16-01742-s001.zip › Table S5.pdf]

Ranch	Type	Date Collected	Age (Months)	Sex	Neutralizing Titer (VSNJV)	Neutralizing Titer (VSIV)
Santa Clara del Roble	Serum	11/22/2021	2	M	1:40	0
Santa Clara del Roble	Serum	11/22/2021	3	F	1:40	0
Santa Clara del Roble	Serum	11/22/2021	4	F	1:40	0
Santa Clara del Roble	Serum	11/22/2021	4	F	0	0
Santa Clara del Roble	Serum	11/22/2021	6	F	1:8	0
Santa Clara del Roble	Serum	11/22/2021	7	M	1:1000	0
Santa Clara del Roble	Serum	11/22/2021	7	M	0	0
Santa Clara del Roble	Serum	11/22/2021	7	F	0	0
Santa Clara del Roble	Serum	11/22/2021	7	M	0	0
Santa Clara del Roble	Serum	11/22/2021	8	M	1:40	0
Santa Clara del Roble	Serum	11/22/2021	10	M	1:8	0
Santa Clara del Roble	Serum	11/22/2021	12	F	1:200	0
Santa Clara del Roble	Serum	11/22/2021	12	F	1:40	0
Santa Clara del Roble	Serum	11/22/2021	12	M	1:200	0
Santa Clara del Roble	Serum	11/22/2021	13	M	1:40	0
Santa Clara del Roble	Serum	11/22/2021	13	M	1:40	0
Santa Clara del Roble	Serum	11/22/2021	14	F	1:200	0
Santa Clara del Roble	Serum	11/22/2021	42	F	1:200	0
Santa Clara del Roble	Serum	11/22/2021	44	F	1:40	0
Santa Clara del Roble	Serum	11/22/2021	45	F	1:1000	0
Santa Clara del Roble	Serum	11/22/2021	56	F	1:40	0
Santa Clara del Roble	Serum	11/22/2021	64	F	1:200	0
Santa Clara del Roble	Serum	11/22/2021	68	F	1:200	0
Santa Clara del Roble	Serum	11/22/2021	69	F	1:5000	0
Santa Clara del Roble	Serum	11/22/2021	70	F	1:5000	0
Santa Clara del Roble	Serum	11/22/2021	79	F	1:1000	1:8
Santa Clara del Roble	Serum	11/22/2021	84	F	1:1000	0
Santa Clara del Roble	Serum	11/22/2021	88	F	1:200	1:8
Santa Clara del Roble	Serum	11/22/2021	92	F	1:200	0

Santa Clara del Roble	Serum	11/22/2021	92	F	1:1000	0
Santa Clara del Roble	Serum	11/22/2021	115	F	1:40	0
Santa Clara del Roble	Serum	11/22/2021	136	F	1:200	0
Kikapu	Serum	11/30/2021	ADULT	F	1:1000	0
Kikapu	Serum	11/30/2021	ADULT	F	1:1000	0
Kikapu	Serum	11/30/2021	ADULT	F	1:40	0
Kikapu	Serum	11/30/2021	ADULT	F	1:1000	0
Kikapu	Serum	11/30/2021	ADULT	F	1:1000	0
Kikapu	Serum	11/30/2021	ADULT	F	1:1000	0
Kikapu	Serum	11/30/2021	ADULT	F	1:1000	1:8
Kikapu	Serum	11/30/2021	ADULT	F	1:40	0
Kikapu	Serum	11/30/2021	ADULT	F	1:5000	1:8
Kikapu	Serum	11/30/2021	ADULT	F	1:5000	1:8
Kikapu	Serum	11/30/2021	ADULT	F	1:5000	0
Kikapu	Serum	11/30/2021	ADULT	F	1:5000	0
Kikapu	Serum	11/30/2021	ADULT	F	1:1000	0
Kikapu	Serum	11/30/2021	ADULT	F	1:5000	0
Kikapu	Serum	11/30/2021	ADULT	F	1:5000	0
Kikapu	Serum	11/30/2021	ADULT	F	1:40	0
Kikapu	Serum	11/30/2021	JUVENILE	F	1:1000	0
Kikapu	Serum	11/30/2021	JUVENILE	F	1:1000	0
Kikapu	Serum	11/30/2021	JUVENILE	M	1:5000	0
Kikapu	Serum	11/30/2021	JUVENILE	M	1:1000	0
Kikapu	Serum	11/30/2021	JUVENILE	F	1:200	0
Kikapu	Serum	11/30/2021	JUVENILE	F	1:5000	0
Kikapu	Serum	11/30/2021	JUVENILE	F	0	0
Kikapu	Serum	11/30/2021	JUVENILE	F	1:8	0
Kikapu	Serum	11/30/2021	JUVENILE	M	0	0
Kikapu	Serum	11/30/2021	JUVENILE	F	1:8	0
Kikapu	Serum	11/30/2021	JUVENILE	F	1:1000	0
Kikapu	Serum	11/30/2021	JUVENILE	M	0	0

Kikapu	Serum	11/30/2021	JUVENILE	M	1:8	0
Kikapu	Serum	11/30/2021	JUVENILE	F	0	0
Kikapu	Serum	11/30/2021	JUVENILE	F	1:8	0
Kikapu	Serum	11/30/2021	JUVENILE	F	1:8	0
El Yaqui	Serum	12/7/2021	0.07	F	1:40	0
El Yaqui	Serum	12/7/2021	4	F	0	0
El Yaqui	Serum	12/7/2021	5	F	1:8	0
El Yaqui	Serum	12/7/2021	5	M	1:40	0
El Yaqui	Serum	12/7/2021	5	M	1:40	0
El Yaqui	Serum	12/7/2021	5	M	1:8	0
El Yaqui	Serum	12/7/2021	5	M	1:8	0
El Yaqui	Serum	12/7/2021	5	M	1:40	0
El Yaqui	Serum	12/7/2021	5	M	0	1:40
El Yaqui	Serum	12/7/2021	5	F	1:8	0
El Yaqui	Serum	12/7/2021	5	F	1:40	0
El Yaqui	Serum	12/7/2021	5	F	0	0
El Yaqui	Serum	12/7/2021	5	F	1:40	0
El Yaqui	Serum	12/7/2021	5	F	1:8	0
El Yaqui	Serum	12/7/2021	6	M	1:8	0
El Yaqui	Serum	12/7/2021	10	F	0	0
El Yaqui	Serum	12/7/2021	24	F	1:200	0
El Yaqui	Serum	12/7/2021	24	F	1:200	0
El Yaqui	Serum	12/7/2021	36	F	1:8	0
El Yaqui	Serum	12/7/2021	36	F	1:200	0
El Yaqui	Serum	12/7/2021	48	F	1:1000	0
El Yaqui	Serum	12/7/2021	48	F	1:200	0
El Yaqui	Serum	12/7/2021	48	F	1:200	0
El Yaqui	Serum	12/7/2021	60	F	1:200	0
El Yaqui	Serum	12/7/2021	72	F	1:1000	0
El Yaqui	Serum	12/7/2021	72	F	1:1000	0
El Yaqui	Serum	12/7/2021	72	F	1:1000	0

El Yaqui	Serum	12/7/2021	72	F	1:1000	0
El Yaqui	Serum	12/7/2021	108	F	1:200	0
El Yaqui	Serum	12/7/2021	108	F	1:1000	0
El Yaqui	Serum	12/7/2021	120	F	1:200	0
El Yaqui	Serum	12/7/2021	120	F	1:1000	0
Veinte Casas	Serum	12/14/2021	4	F	1:1000	0
Veinte Casas	Serum	12/14/2021	5	M	1:5000	0
Veinte Casas	Serum	12/14/2021	6	F	1:5000	0
Veinte Casas	Serum	12/14/2021	6	M	1:25000	0
Veinte Casas	Serum	12/14/2021	7	M	1:1000	0
Veinte Casas	Serum	12/14/2021	8	M	1:1000	0
Veinte Casas	Serum	12/14/2021	8	F	1:1000	0
Veinte Casas	Serum	12/14/2021	8	F	0	0
Veinte Casas	Serum	12/14/2021	8	F	0	1:8
Veinte Casas	Serum	12/14/2021	9	M	0	0
Veinte Casas	Serum	12/14/2021	10	F	1:200	0
Veinte Casas	Serum	12/14/2021	12	M	0	0
Veinte Casas	Serum	12/14/2021	12	F	0	1:8
Veinte Casas	Serum	12/14/2021	14	F	0	0
Veinte Casas	Serum	12/14/2021	36	F	0	0
Veinte Casas	Serum	12/14/2021	48	F	1:5000	1:8
Veinte Casas	Serum	12/14/2021	48	M	1:200	0
Veinte Casas	Serum	12/14/2021	60	F	1:40	0
Veinte Casas	Serum	12/14/2021	72	F	1:5000	0
Veinte Casas	Serum	12/14/2021	72	F	1:5000	1:8
Veinte Casas	Serum	12/14/2021	72	F	1:1000	0
Veinte Casas	Serum	12/14/2021	72	F	1:1000	0
Veinte Casas	Serum	12/14/2021	84	F	1:1000	0
Veinte Casas	Serum	12/14/2021	84	F	1:1000	0
Veinte Casas	Serum	12/14/2021	84	F	0	0
Veinte Casas	Serum	12/14/2021	84	F	1:200	0

Veinte Casas	Serum	12/14/2021	84	F	1:1000	0
Veinte Casas	Serum	12/14/2021	96	F	1:1000	1:8
Veinte Casas	Serum	12/14/2021	96	F	1:5000	0
Veinte Casas	Serum	12/14/2021	96	F	1:8	0
Veinte Casas	Serum	12/14/2021	120	F	1:1000	1:8
Veinte Casas	Serum	12/14/2021	144	M	1:200	0
Raudal del Potro	Serum	12/21/2021	4	M	1:40	0
Raudal del Potro	Serum	12/21/2021	6	F	0	0
Raudal del Potro	Serum	12/21/2021	7	F	0	0
Raudal del Potro	Serum	12/21/2021	7	F	0	0
Raudal del Potro	Serum	12/21/2021	7	F	0	0
Raudal del Potro	Serum	12/21/2021	7	F	0	0
Raudal del Potro	Serum	12/21/2021	7	F	1:8	0
Raudal del Potro	Serum	12/21/2021	7	M	0	0
Raudal del Potro	Serum	12/21/2021	7	F	0	0
Raudal del Potro	Serum	12/21/2021	7	M	0	0
Raudal del Potro	Serum	12/21/2021	8	M	0	0
Raudal del Potro	Serum	12/21/2021	12	F	1:200	0
Raudal del Potro	Serum	12/21/2021	12	M	1:200	0
Raudal del Potro	Serum	12/21/2021	20	F	1:1000	0
Raudal del Potro	Serum	12/21/2021	21	F	1:1000	0
Raudal del Potro	Serum	12/21/2021	24	F	1:5000	0
Raudal del Potro	Serum	12/21/2021	24	F	1:5000	0
Raudal del Potro	Serum	12/21/2021	24	F	1:1000	0
Raudal del Potro	Serum	12/21/2021	24	F	1:1000	0
Raudal del Potro	Serum	12/21/2021	24	F	1:1000	1:8
Raudal del Potro	Serum	12/21/2021	24	F	1:5000	0
Raudal del Potro	Serum	12/21/2021	24	F	1:1000	0
Raudal del Potro	Serum	12/21/2021	36	F	1:5000	0
Raudal del Potro	Serum	12/21/2021	36	F	1:5000	0
Raudal del Potro	Serum	12/21/2021	36	F	1:1000	0

Raudal del Potro	Serum	12/21/2021	48	F	1:1000	0
Raudal del Potro	Serum	12/21/2021	60	F	1:5000	1:8
Raudal del Potro	Serum	12/21/2021	72	F	1:1000	0
Raudal del Potro	Serum	12/21/2021	84	F	1:5000	0
Raudal del Potro	Serum	12/21/2021	96	F	1:5000	0
Raudal del Potro	Serum	12/21/2021	108	M	1:1000	0
Raudal del Potro	Serum	12/21/2021	156	F	1:1000	1:8
