# Supplementary material for: Vesicular Stomatitis Virus Transmission Dynamics Within Its Endemic Range in Chiapas, Mexico
Source: Viruses. 2024 Nov 6;16(11):1742. doi: 10.3390/v16111742 (PMC11598859; doi:10.3390/v16111742)
Supplement: Supplementary file 1 [file viruses-16-01742-s001.zip › Table S6.pdf]

Psychodidae

	***Lutzomyia cruciata* (n =5)**	
	To GenBank Sequence	Between Samples
Min	97.81%	98.62%
Max	99.15%	99.63%
Avg	99.50%	99.11%
± SD	0.01%	0.31%

	***Lutzomyia shannoni* (n =5)**	
	To GenBank Sequence	Between Samples
Min	98.40%	NA
Max	98.40%	NA
Avg	98.40%	NA
± SD	NA	NA

	***Lutzomyia evansi* (n =7)**	
	To GenBank Sequence	Between Samples
Min	98.69%	99.90%
Max	98.81%	100.00%
Avg	98.76%	99.97%
± SD	0.05%	0.03%

	***Lutzomyia spp.* (n =2)**	
	To GenBank Sequence (*Lutzomyia cruciata*)	Between Samples
Min	92.15%	99.35%
Max	92.78%	99.35%
Avg	92.47%	99.35%
± SD	0.21%	NA

	***Psathyromyia maya* (n =1)**	
	To GenBank Sequence	Between Samples
Min	96.95%	NA
Max	96.95%	NA
Avg	96.95%	NA
± SD	NA	NA

	Lutzomyia (Dampfomyia) sp. (n =1)	
	To GenBank Sequence (*Dampfomyia anthrophora*)	Between Samples
Min	90.13%	NA
Max	90.13%	NA
Avg	90.13%	NA
± SD	NA	NA

	Psychoda alternata (n =10)	
	To GenBank Sequences	Between Samples
Min	99.67%	99.70%
Max	100.00%	100.00%
Avg	99.87%	100.00%
± SD	0.10%	0.08%

	Clogmia (Psychoda) albipunctata (n =2)	
	To GenBank Sequence	Between Samples
Min	99.58%	99.58%
Max	100.00%	99.58%
Avg	99.79%	99.58%
± SD	0.21%	NA

	Psychoda spp. (n =2)	
	To GenBank Sequences (Psychoda cinerea)	Between Samples
Min	88.29%	99.83%
Max	88.50%	99.83%
Avg	88.40%	99.83%
± SD	0.10%	NA

Ceratopogonidae

	Culicoides insignis (n =4)	
	To GenBank Sequence	Between Samples
Min	96.49%	99.65%
Max	96.68%	100.00%
Avg	96.63%	99.72%
± SD	0.13%	0.13%

	Culicoides neopulicaris **(n =1)**	
	To GenBank Sequence	Between Samples
Min	99.74%	NA
Max	99.74%	NA
Avg	99.74%	NA
± SD	NA	NA

	Culicoides hylas **(n =3)**	
	To GenBank Sequence	Between Samples
Min	98.48%	98.99%
Max	98.62%	99.62%
Avg	98.55%	99.29%
± SD	0.07%	0.40%

	Culicoides bambusicola **(n =1)**	
	To GenBank Sequence	Between Samples
Min	99.56%	NA
Max	99.56%	NA
Avg	99.56%	NA
± SD	NA	NA

	Culicoides sp. **(n =1)**	
	To GenBank Sequence (*Culicoides diabolicus*)	Between Samples
Min	87.44%	NA
Max	87.44%	NA
Avg	87.44%	NA
± SD	NA	NA

	Ceratopogonidae sp. **(n =1)**	
	To GenBank Sequence (*Culicoides furens*)	Between Samples
Min	86.13%	NA
Max	86.13%	NA
Avg	86.13%	NA
± SD	NA	NA

Culex

	***Aedes (Ochlerotatus) angustivittatus* (n = 4)**	
	To GenBank Sequence	Between Samples
Min	99.41%	99.41%
Max	99.82%	99.72%
Avg	99.55%	99.55%
± SD	0.15%	0.14%

	***Aedes guatemala* (n = 2)**	
	To GenBank Sequence	Between Samples
Min	99.42%	99.82%
Max	99.82%	99.82%
Avg	99.62%	99.82%
± SD	0.11%	N/A

	***Mansonia titillans* (n = 2)**	
	To GenBank Sequence	Between Samples
Min	99.50%	99.50%
Max	100.00%	99.50%
Avg	99.75%	99.50%
± SD	0.25%	N/A

	***Mansonia dyari* (n = 3)**	
	To GenBank Sequence	Between Samples
Min	97.67%	98.93%
Max	98.51%	99.92%
Avg	98.22%	99%
± SD	0.38%	0.44%

	***Psorophora horrida* (n = 2)**	
	To GenBank Sequence	Between Samples
Min	99.48%	100%
Max	99.51%	100%
Avg	99.50%	100%
± SD	0.01%	N/A

	Culex spp. **(n = 34)**	
	To GenBank Sequence (*C. bidens*)	Between Samples
Min	97.86%	97.62%
Max	99.18%	100.00%
Avg	98.43%	99.53%
± SD	0.23%	0.61%

	Culex spp. **(n = 34)**	
	To GenBank Sequence (*C. nigripalpus*)	Between Samples
Min	97.25%	97.62%
Max	99.02%	100.00%
Avg	98.04%	99.53%
± SD	0.41%	0.61%

	Culex spp. **(n = 34)**	
	To GenBank Sequence (*C. conspirator*)	Between Samples
Min	97.85%	97.62%
Max	99.59%	100.00%
Avg	98.17%	99.53%
± SD	0.36%	0.61%

	Culex spp. **(n = 34)**	
	To GenBank Sequence (*C. declarator*)	Between Samples
Min	96.93%	97.62%
Max	99.34%	100.00%
Avg	97.68%	99.53%
± SD	0.49%	0.61%

	***Culex spp.* (n = 34)**	
	To GenBank Sequence (*C. lactator*)	Between Samples
Min	97.13%	97.62%
Max	99.18%	100.00%
Avg	97.51%	99.53%
± SD	0.53%	0.61%

	***Culex spp.* (n = 34)**	
	To GenBank Sequence (*C. interrogator*)	Between Samples
Min	96.74%	97.62%
Max	99.17%	100.00%
Avg	97.50%	99.53%
± SD	0.49%	0.61%
