# Supplementary figures and images for: Vesicular Stomatitis Virus Transmission Dynamics Within Its Endemic Range in Chiapas, Mexico
Source: Viruses. 2024 Nov 6;16(11):1742. doi: 10.3390/v16111742 (PMC11598859; doi:10.3390/v16111742)

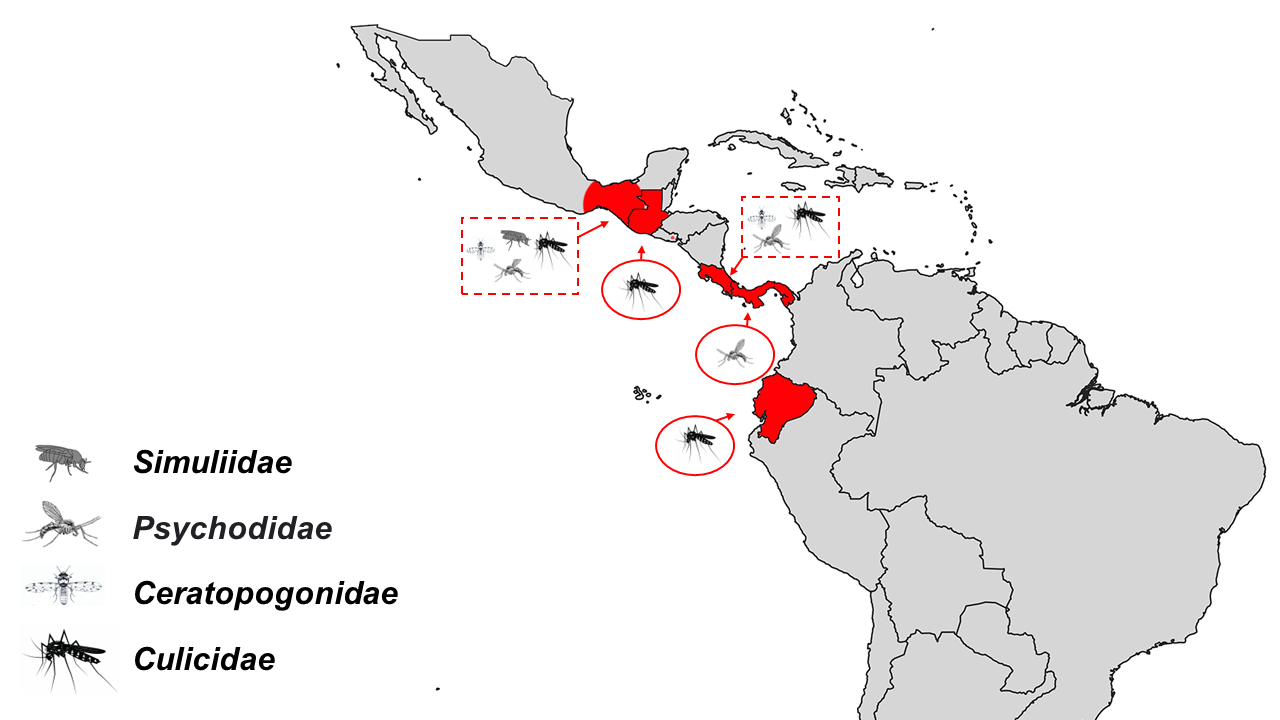

Supplement: Supplementary file 1 [file viruses-16-01742-s001.zip › Figure S1.png]

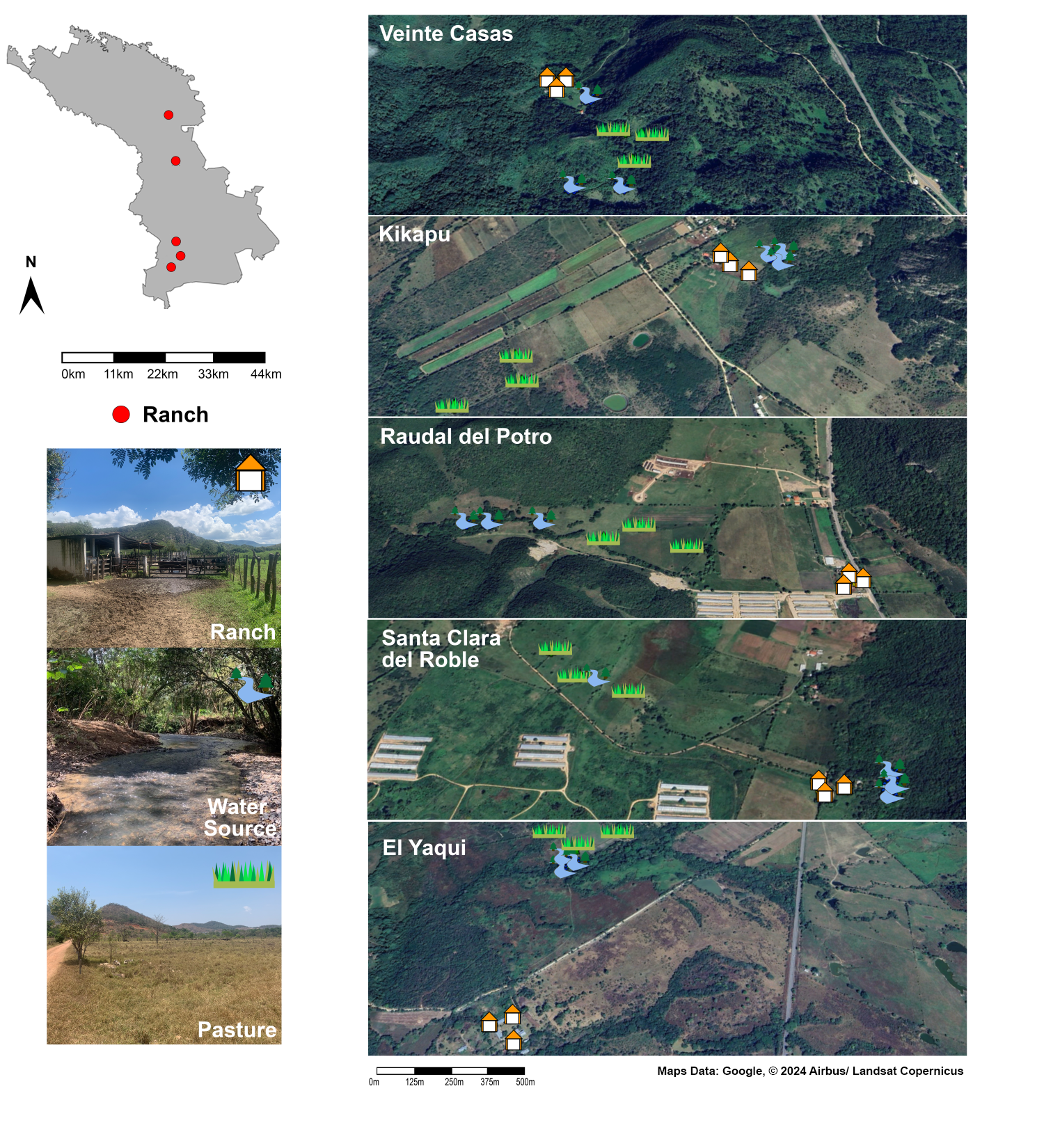

Supplement: Supplementary file 1 [file viruses-16-01742-s001.zip › Figure S2.png]

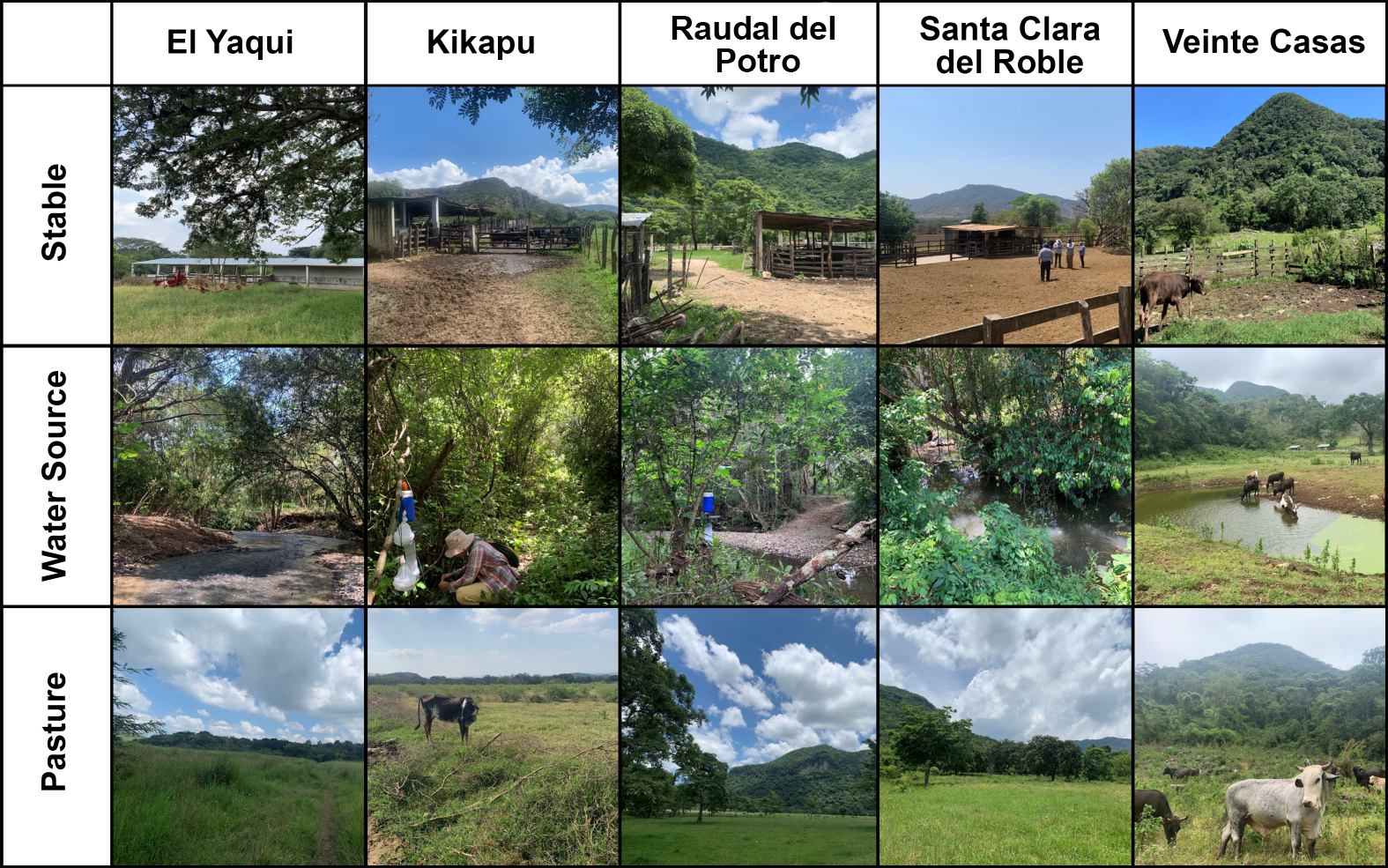

Supplement: Supplementary file 1 [file viruses-16-01742-s001.zip › Figure S3.png]

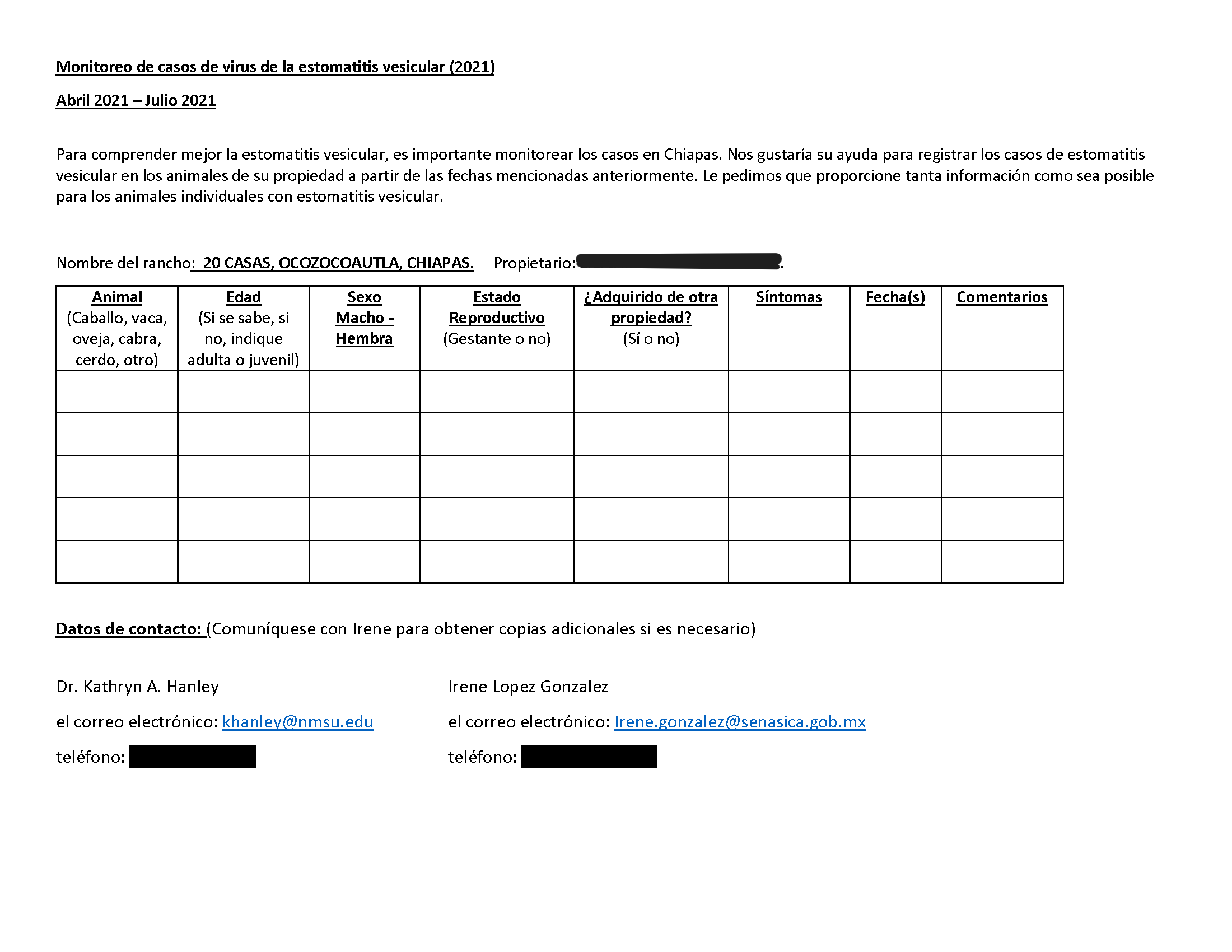

Supplement: Supplementary file 1 [file viruses-16-01742-s001.zip › Figure S4.png]

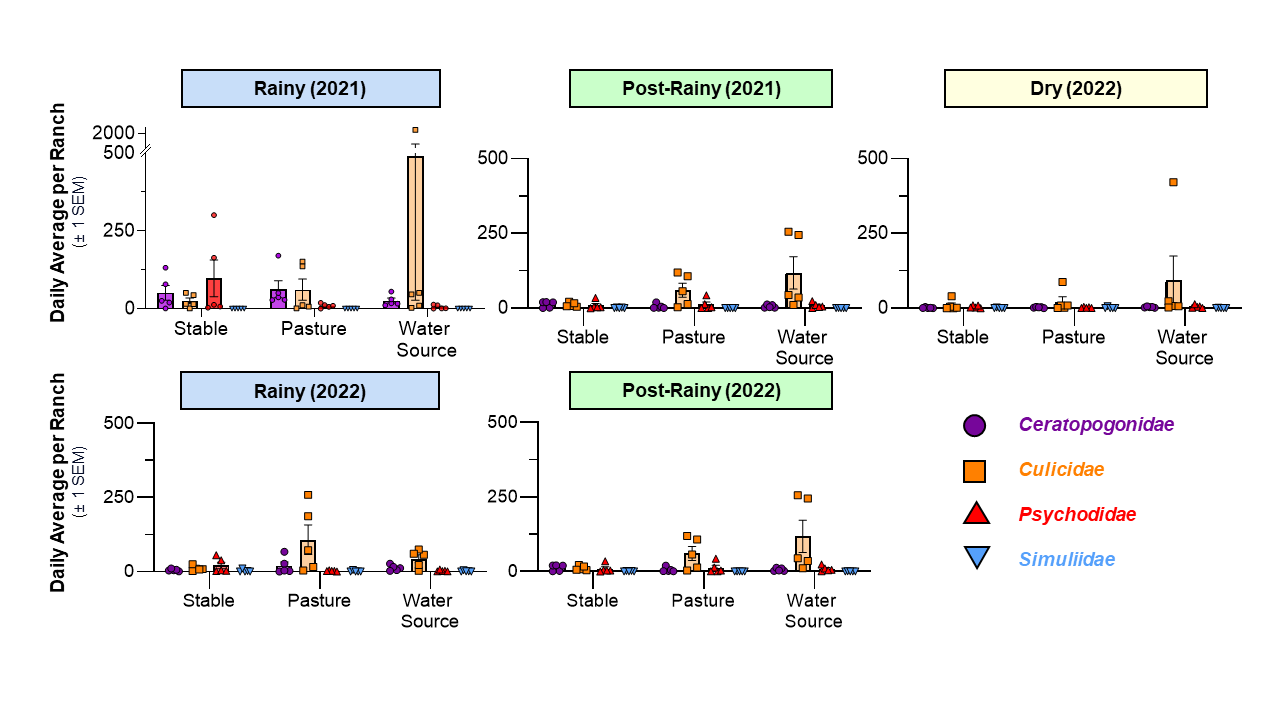

Supplement: Supplementary file 1 [file viruses-16-01742-s001.zip › Figure S5.png]

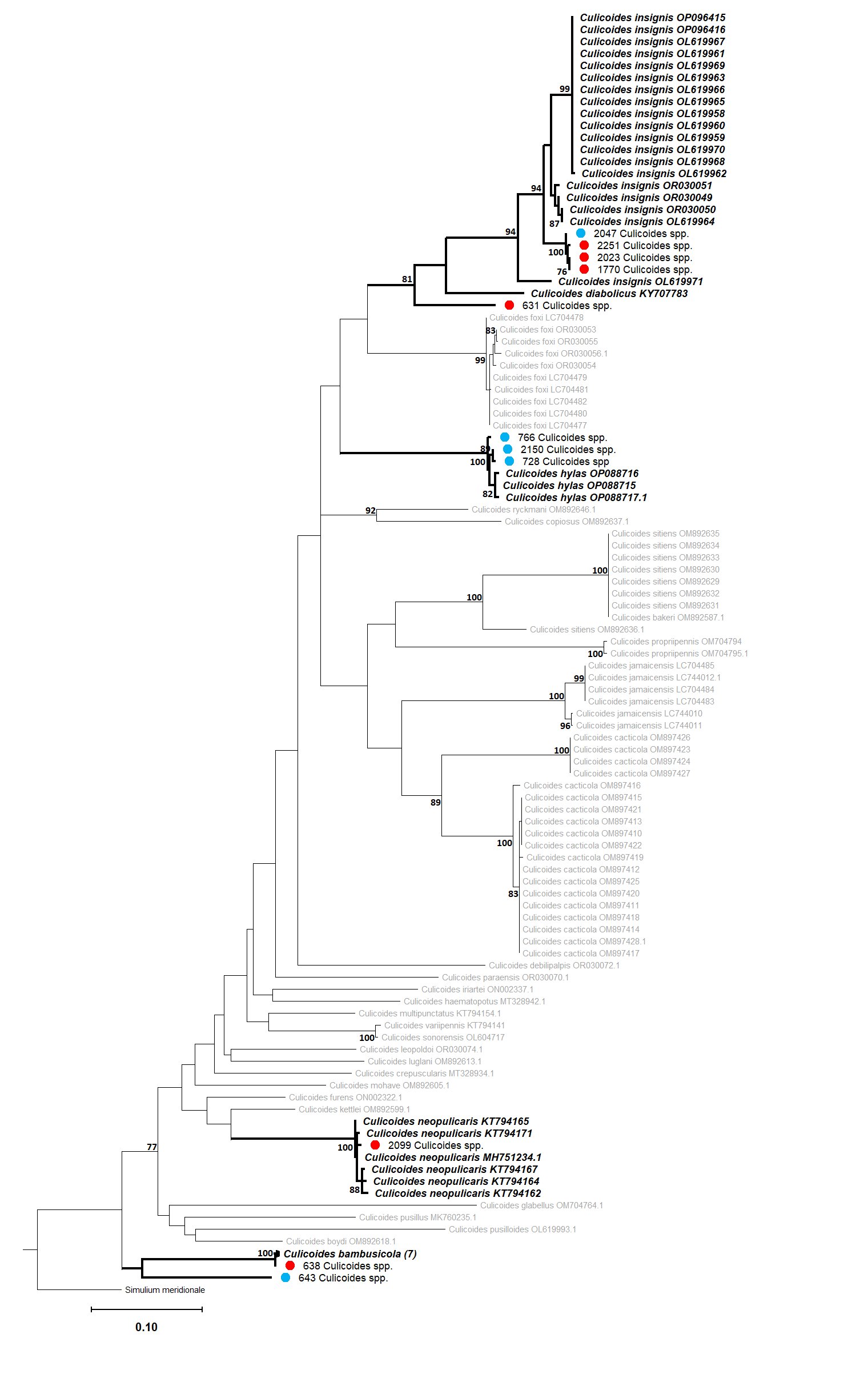

Supplement: Supplementary file 1 [file viruses-16-01742-s001.zip › Figure S6.png]

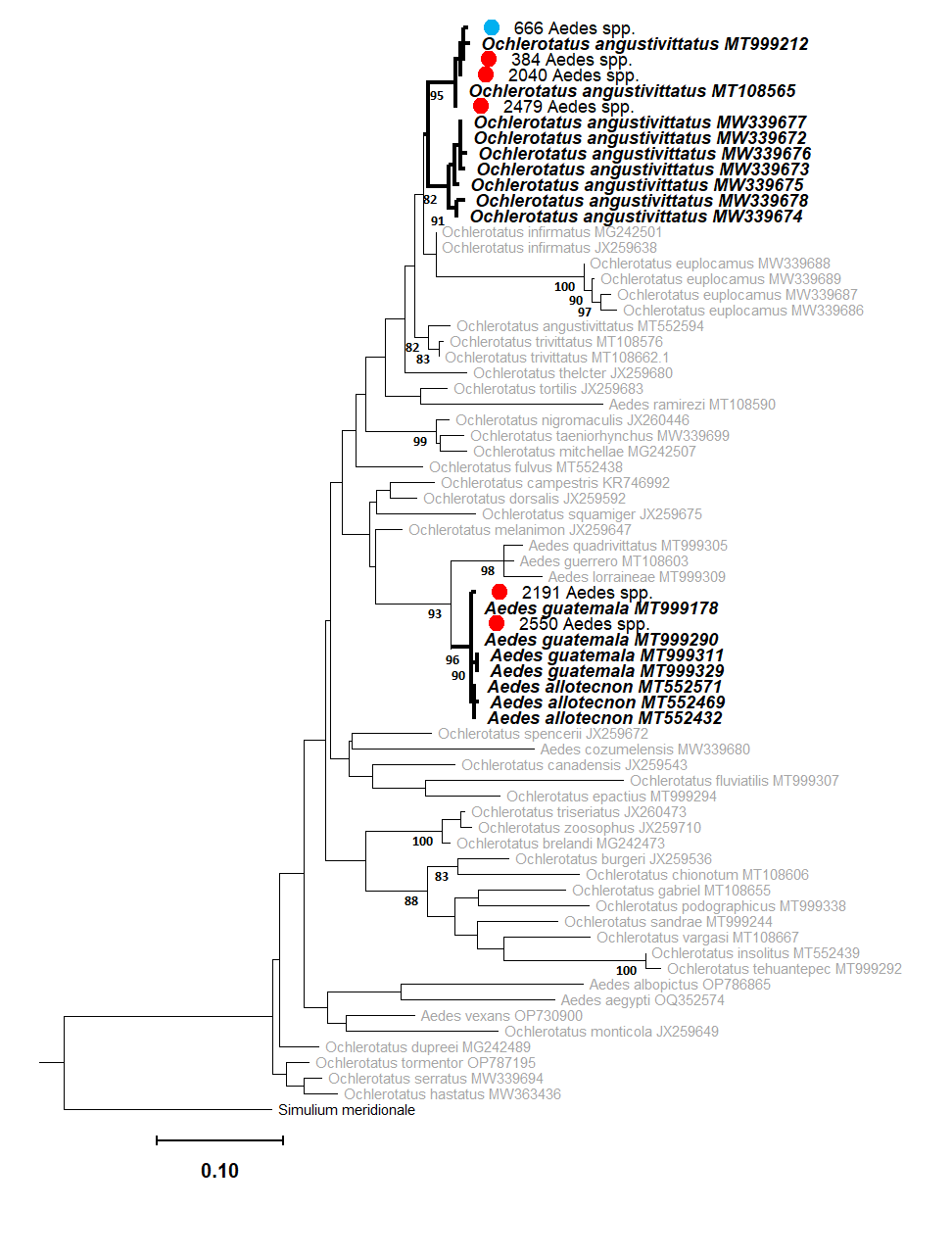

Supplement: Supplementary file 1 [file viruses-16-01742-s001.zip › Figure S7.png]

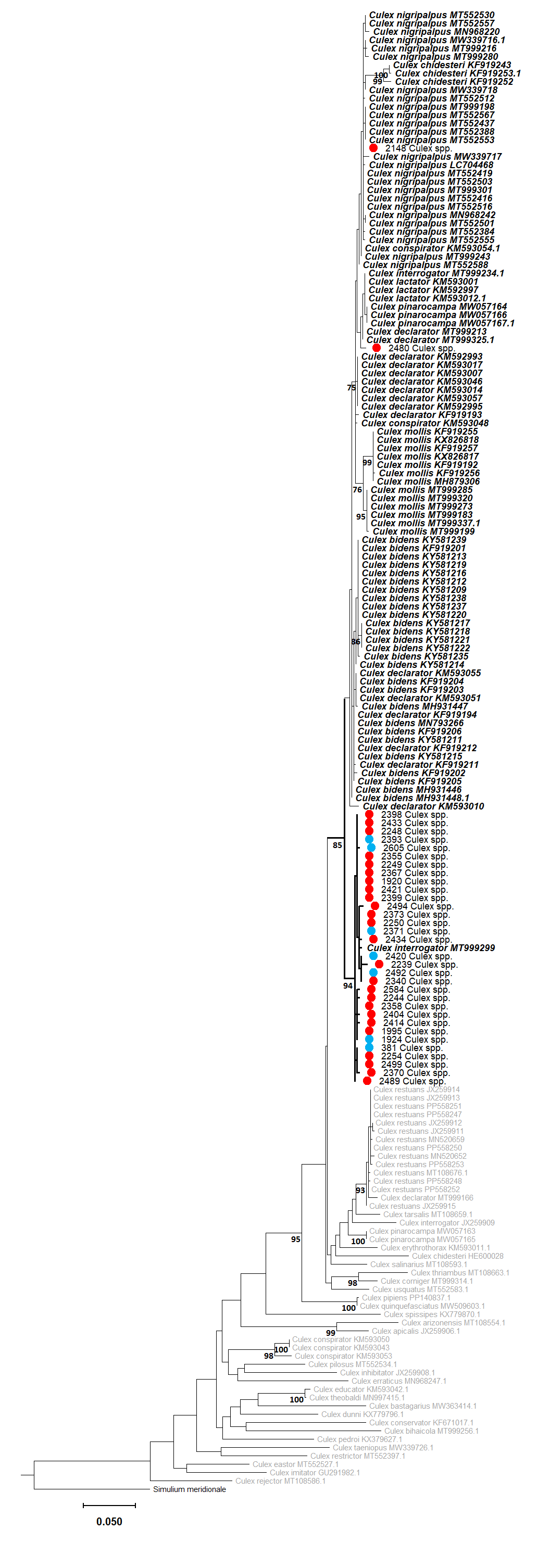

Supplement: Supplementary file 1 [file viruses-16-01742-s001.zip › Figure S8.png]
